# Cell cycle arrest is an important mechanism of action of compound Kushen injection in the prevention of colorectal cancer

**DOI:** 10.1038/s41598-022-08336-4

**Published:** 2022-03-14

**Authors:** Jie Sun, Mei Li, Tingru Lin, Di Wang, Jingyi Chen, Yu Zhang, Qing Mu, Huiting Su, Na Wu, Aiyu Liu, Yimeng Yu, Yulan Liu, Shaojie Wang, Xin Yu, Jingzhu Guo, Weidong Yu

**Affiliations:** 1 Department of Central Laboratory and Institute of Clinical Molecular Biology, Peking University People’s Hospital, Beijing, China; 2 Department of Gastroenterology, Peking University People’s Hospital, Beijing, China; 3 Department of Traditional Chinese Medicine, Peking University People’s Hospital, Beijing, China; 4 Department of Hepatobiliary Surgery, Peking University People’s Hospital, Beijing, China; 5 Department of Pediatric, Peking University People’s Hospital, Beijing, China

**Keywords:** Cancer therapy, Gastrointestinal cancer

## Abstract

Compound Kushen injection (CKI) is the most widely used traditional Chinese medicine preparation for the comprehensive treatment of colorectal cancer (CRC) in China, but its underlying molecular mechanisms of action are still unclear. The present study employed a network pharmacology approach, in which we constructed a “bioactive compound-target-pathway” network. Experimental RNA sequencing (RNA-Seq) analysis was performed to identify a key “bioactive compound-target-pathway” network for subsequent experimental validation. Cell cycle, proliferation, autophagy, and apoptosis assays and a model of azoxymethane/dextran sodium sulfate-induced colorectal carcinogenesis in mice were employed to detect the biological effect of CKI on CRC. Real-time reverse-transcription polymerase chain reaction, Western blot, and immunohistochemistry were performed to verify the selected targets and pathways. We constructed a predicted network that included 82 bioactive compounds, 34 targets, and 33 pathways and further screened an anti-CRC CKI “biological compound (hesperetin 7-*O*-rutinoside, genistein 7-*O*-rutinoside, and trifolirhizin)-target (p53 and checkpoint kinase 1 [CHEK1])” network that targeted the “cell cycle pathway”. Validation experiments showed that CKI effectively induced the cell-cycle arrest of CRC cells in vitro and suppressed the development of CRC in vivo by downregulating the expression of p53 and CHEK1. Our findings confirmed that inducing cell-cycle arrest by CKI is an important mechanism of its anti-CRC action, which provides a direct and scientific experimental basis for the clinical application of CKI.

## Introduction

Colorectal cancer (CRC) is one of the most common human malignant tumors. Its incidence and mortality ranks the third worldwide^1^. China's National Cancer Center reported that the incidence and mortality of CRC in China were third and fifth, respectively, of all malignant tumors in 2019. Most patients are in the middle and late stages of the disease when they are diagnosed^2^. For advanced CRC, in addition to standardized surgery, chemotherapy, radiotherapy, and immunotherapy, traditional Chinese medicine (TCM) is the most important supplementary treatment for CRC. It has been long and widely used in China and shown to suppress the progression of CRC^3–5^, improve the efficacy and reduce the side effects of chemotherapeutic drugs, improve quality of life^6^, and improve prognosis^7–11^.

Compound Kushen injection (CKI) is extracted from Kushen (*Sophorae flavescentis*) and Baituling (Rhizoma Smilacis Glabrae). It has been clinically applied for at least 15 years. It has been shown to have significant curative effects on CRC and is the most commonly used TCM preparation for its comprehensive treatment^9^. Although CKI can inhibit the progression of advanced CRC, enhance the efficacy of chemotherapeutic drugs, reduce the side effects of radiotherapy and chemotherapy, improve quality of life, improve prognosis^6,9^, and inhibit the proliferation, migration, and invasion of CRC cells in vitro^12–14^, its active ingredients, target genes, and molecular pathways by which it inhibits the development of CRC are still unclear. A TCM network pharmacology approach provides a new research paradigm for translating TCM from experience-based medicine to evidence-based medicine, which will likely accelerate TCM discovery and improve drug discovery strategies^15^. A combination of network pharmacology and experimental verification has gradually become an important way to study the mechanism of action of CKI^16–18^.

**Figure 1:**
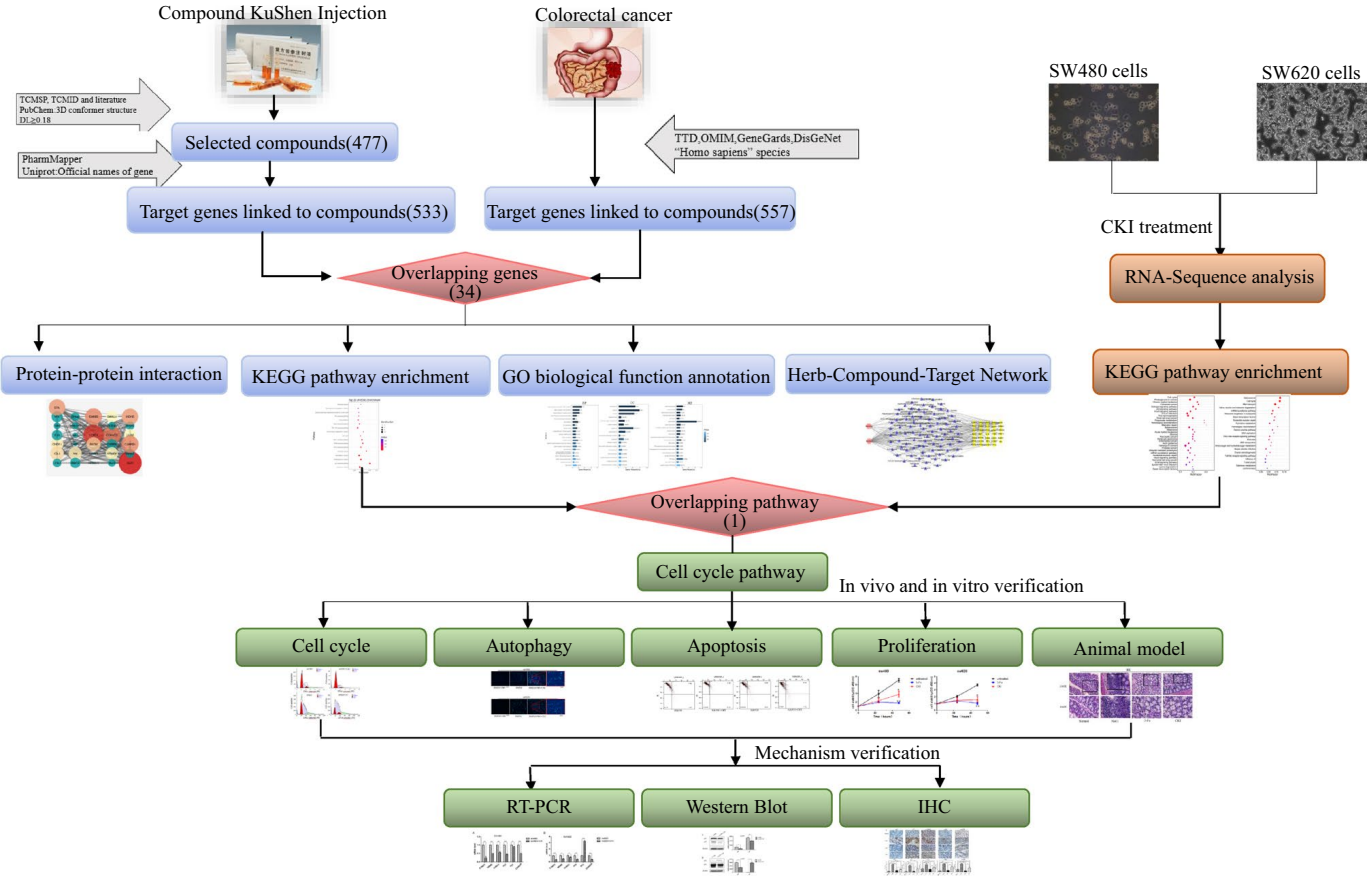
Workflow of network pharmacology and experimental validation analyses.

In the present study, we first used network pharmacology to construct an “herb-bioactive compound-target” network and predict the signaling pathways of CKI for the treatment of CRC. RNA-Seq was then used to screen the key signaling pathway of CKI that affects CRC cells in vitro. Finally, cell and azoxymethane/dextran sodium sulfate (AOM/DSS) animal models were used to further verify the mechanism of CKI in inhibiting CRC by targeting this key pathway. Figure 1 depicts a flowchart of the technical strategy that was used in this study.

## Results

### Screening of bioactive compounds in CKI and related targets

We first screened a total of 477 active components in CKI, of which 325 were unique to Kushen, 109 were unique to Baituling, and 43 were common to both. A total of 533 related target genes of these 477 chemical ingredients were then predicted for subsequent analysis (Fig. 1).

### Prediction of anti-CRC targets of bioactive compounds in CKI

**Figure 2:**
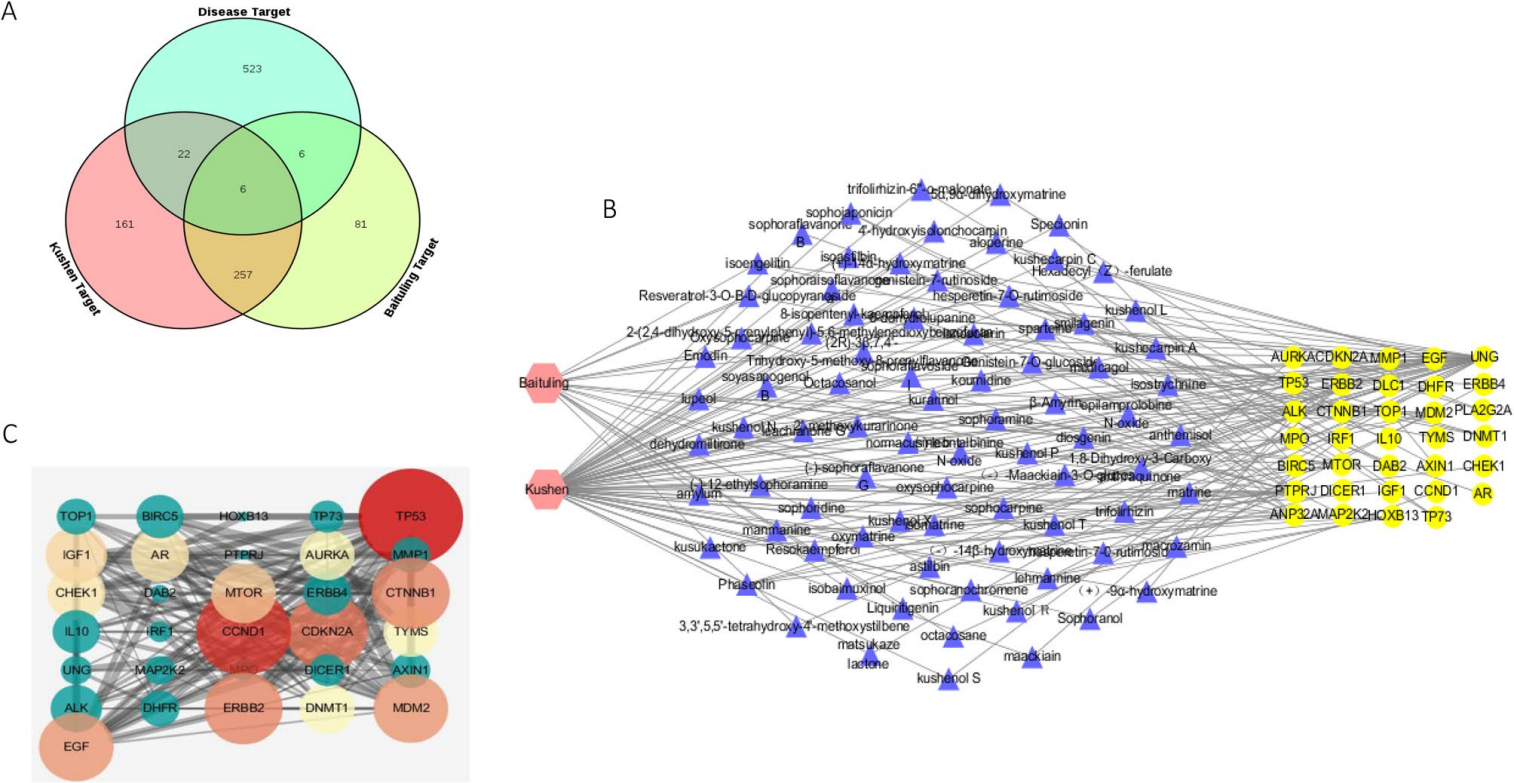
Network pharmacological analysis of targets, networks, and PPI. (**A**) Venn diagram of potential targets of CKI for CRC. The analysis and image was performed using the OmicShare tools, a free online platform for data analysis. (**B**) “Herb-active ingredient-protein target” network for CKI. The pink nodes represent herbs in CKI. Blue nodes represent potential drug targets. Yellow nodes represent active compounds. The edges represent interactions among them. The image was created using Cytoscape 3.7.1 software. (**C**) Protein interaction network of targets of CKI for the treatment of CRC. Each node represents the relevant gene. The edge means line thickness, which indicates the strength of data support. Hubs of top 14 genes in the PPI network are expressed in colors other than green. The depth of the colors and the size of the circle represent the degree. The image was created using STRING 10.5 database.

A total of 644 target genes that are related to CRC were collected from the TTD (Therapeutic Target Database) (84 genes), OMIM (Online Mendelian Inheritance in Man) (197 genes), GeneCards (200 genes), and DisGeNET (a database of gene-disease associations) (163 genes) databases. After integrating and deleting duplicate genes, 557 of 644 target genes were selected to merge the 533 target genes that are related to bioactive compounds in CKI. Finally, we obtained 34 candidate CRC target genes, corresponding to 82 bioactive compounds in CKI (Fig. 2A,B, Supplementary Table S1). Among these, *TP53*, *MDM2*, *ERBB2*, *HOXB13*, *UNG*, and *PTPRJ* are common targets of Kushen and Baituling, *CHEK1*, *TP73*, *MTOR*, *IRF1*, *IL10*, *CTNNB1*, *CCND1*, *EGF*, *MAP2K2*, *ANP32A*, *IGF1*, *AXIN1*, *DAB2*, *BIRC5*, *PLA2G2A*, *ALK*, *AURKA*, *DHFR*, *MPO*, *TYMS*, *AR*, and *TOP1* are unique targets of Kushen, and *DNMT1*, *ERBB4*, *DICER1*, *DLC1*, *CDKN2A*, and *MMP1* are unique targets of Baituling (Supplementary Table S1).

### Analysis of target protein–protein interaction (PPI) network

As shown in Fig. 2C, the visual PPI network, which included 31 nodes and 174 edges, was obtained after removing targets that are independent of the network. Node size represents the degree value, in which a larger node indicates a greater degree value. The thickness of the side represents the combined score, in which a thicker side indicates a stronger interaction between proteins. In the present study, 14 target proteins with a degree value greater than the average were used as hub target proteins (Supplementary Table S2), such as TP53, CCND1, CDKN2A, MDM2, CTNNB1, and CHEK1.

### Construction and analysis of “herb-bioactive compound-target” network

As shown in Fig. 2B, the “herb-compound-target” network, which was constructed from 82 bioactive compounds and 34 candidate CRC target genes, included 118 nodes and 402 edges, with a network density of 0.030 and network diameter of 8. Detailed information about this network is shown in Supplementary Table S3. From this predicted network, we found that one bioactive compound (trifolirhizin) acts on multiple target genes, and one target gene (*p53*) can also be regulated by multiple bioactive compounds (i.e., genistein-7-rutinoside, lanceolarin, and hesperetin-7-*O*-rutimoside).

### Gene Ontology (GO) biological function annotation and Kyoto Encyclopedia of Genes and Genomes (KEGG) pathway enrichment analysis of targets

**Figure 3:**
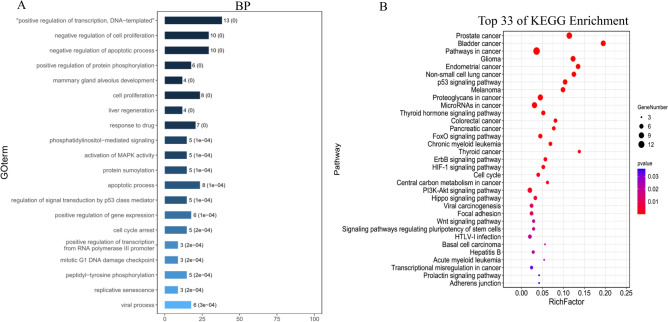
Functional analysis of identified compound-related targets. (**A**) Enrichment analysis of Gene Ontology (GO) biological processes of anti-cancer target genes of active ingredients of CKI. (**B**) Kyoto Encyclopedia of Genes and Genomes (KEGG) pathways related to anti-cancer targets of CKI. The image was created using the OmicShare tools, a free online platform for data analysis.

A total of 34 potential CRC target genes were used to perform GO biological functional annotation and KEGG pathway enrichment analyses. As shown in Fig. 3A, the top 20 enriched GO terms (adjusted *p* < 0.05) in the Biological Processes category suggested that these genes are strongly associated with multiple biological processes, such as cell cycle arrest, cell proliferation, apoptotic process, regulation of signal transduction by p53 class mediator, pathway, mitotic G1 DNA damage checkpoint, and so on. As shown in Fig. 3B, 33 enriched KEGG pathways (*p* < 0.05) are mainly involved in the cell cycle, p53 signaling pathway, PI3K-Akt signaling pathway, CRC, proteoglycans in cancer, pathways in cancer, and so on (Supplementary Table S4). Integrating the results of the bioactive compound screening and CRC-specific target genes from the GO annotation and KEGG pathway analyses, we successfully constructed a prediction network of anti-CRC CKI that comprised 82 active compounds, 34 protein targets, and 33 pathways (Figs. 2, 3).

### Experimental RNA-Seq validation of predicted KEGG pathways and selection of common “bioactive compound-target-pathway” network of anti-CRC CKI

**Figure 4:**
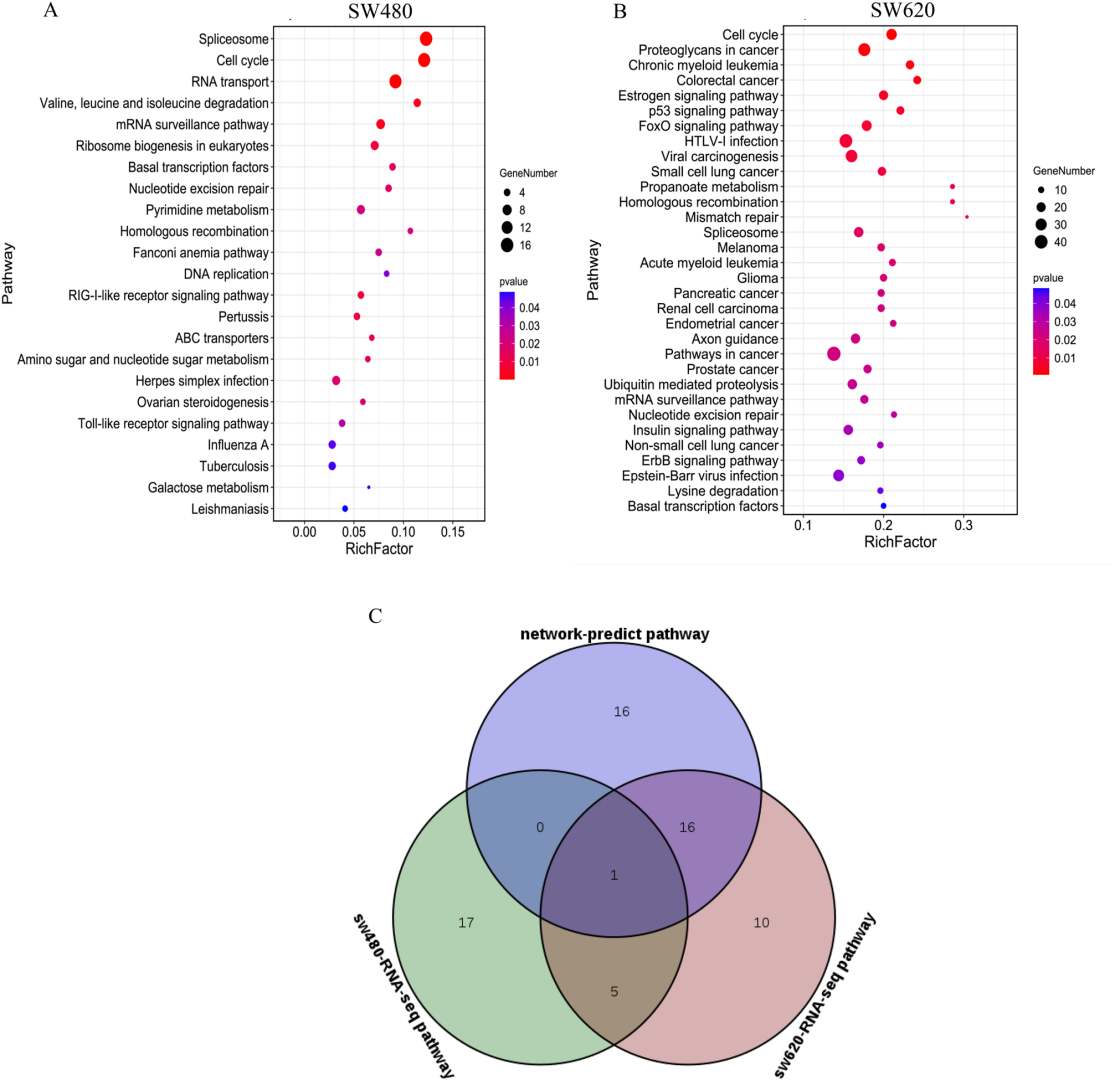
Functional analysis of CKI against SW480 and SW620 cells based on RNA-seq data. (**A**) Kyoto Encyclopedia of Genes and Genomes (KEGG) pathways related to CKI against SW480 cells. (**B**) KEGG pathways related to CKI against SW620 cells. (**C**) Venn diagram of pathways that were enriched based on RNA-Seq data and predicted by network pharmacology. The image was created using the OmicShare tools, a free online platform for data analysis.

To verify the KEGG pathways of anti-CRC CKI that were predicted above, RNA-Seq was performed to detect the global differential gene expression profile and enrich the KEGG pathways of SW480 or SW620 CRC cells with and without CKI treatment. As shown in Fig. 4A,B, 23 and 32 KEGG pathways (Supplementary Table S4) were enriched from SW480 and SW620 cells, respectively. Seventeen KEGG enrichment pathways in SW620 cells were the same as predicted by the above network pharmacology, whereas only one such pathway in SW480 cells was identified, namely the “cell cycle” pathway, which was a common pathway of the three (network-predict pathway, SW480-RNA-Seq pathway and SW620-RNA-Seq pathway**)** (Fig. 4C) and also a key pathway for anti-CRC CKI.

### Compound Kushen injection induces cell cycle arrest of SW480 and SW620 CRC cells in vitro

**Figure 5:**
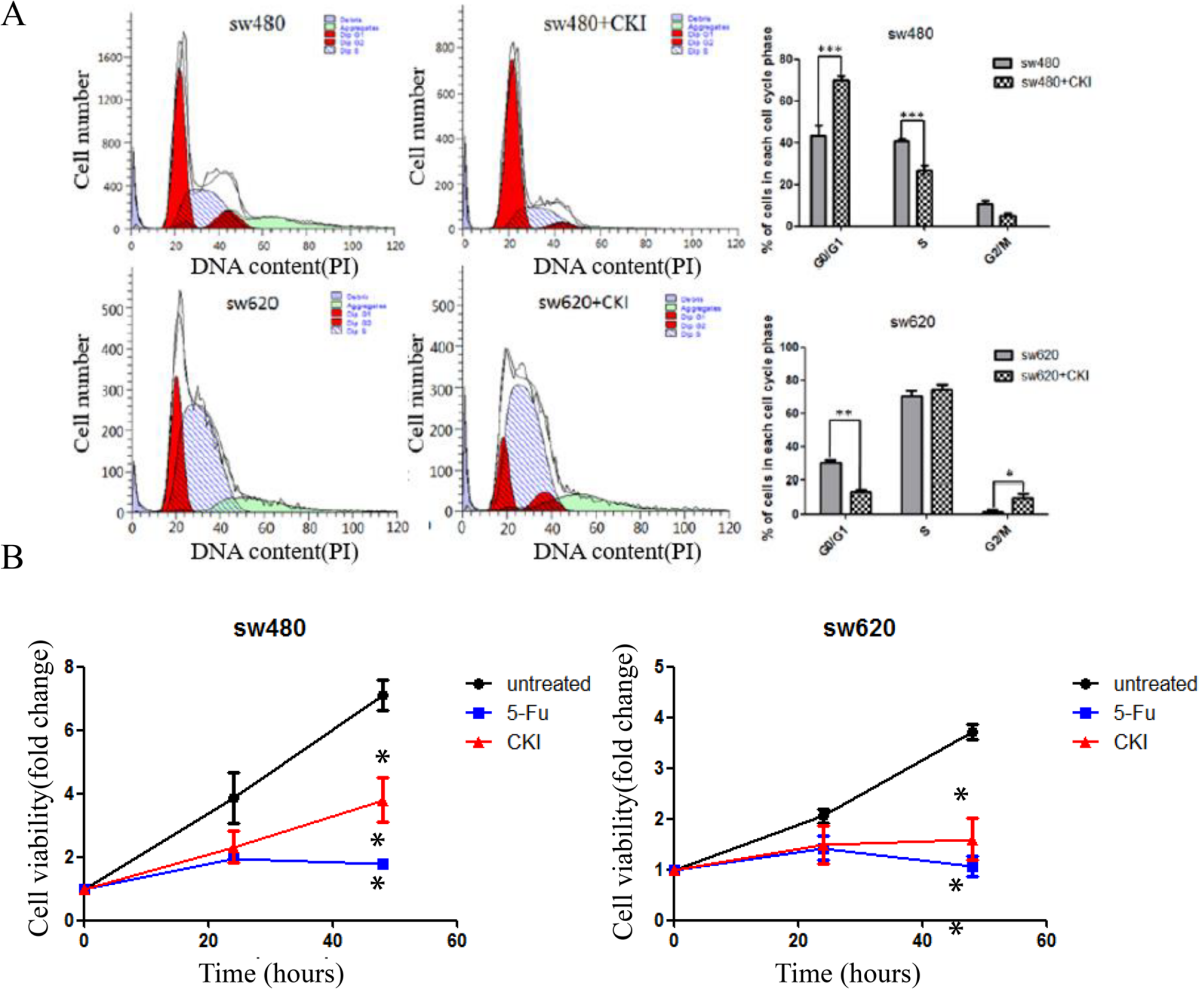
Compound Kushen injection induces the arrest of cell cycle progression and inhibits CRC cell proliferation in vitro. (**A**) Flow cytometry assays were performed to analyze the cell cycle in SW480 and SW620 cells after CKI treatment. Values at different stages of the cell cycle represent the mean ± SD from three independent experiments. (**B**) SW480 and SW620 cell viability was measured under different treatment conditions using CCK-8. Each data point represents the mean ± SD from three independent experiments. *p < 0.05, **p < 0.01, ***p < 0.001, compared with control. The image was created using GraphPad Prism 5.01 software.

To characterize the effect of CKI on the cell cycle arrest of SW480 and SW620 CRC cells, both cell lines were labeled with propidium iodide to detect cell cycle progression by flow cytometry. As shown in Fig. 5A, after CKI treatment, the SW480 cell cycle was significantly inhibited in the G0/G1 phase compared with the control (*p* < 0.001), and the SW620 cell cycle was arrested in the G2/M phase (*p* < 0.05).

### Compound Kushen injection inhibits the proliferation of SW480 and SW620 CRC cells in vitro

To determine the effect of CKI on the proliferation of SW480 and SW620 CRC cells, we performed the Cell Counting Kit-8 (CCK-8) assay to measure cell viability after treatment with CKI (2 mg/ml, based on the total alkaloid concentration in CKI). As shown in Fig. 5B, the proliferation of SW480 and SW620 cells was significantly inhibited by treatment with CKI (both *p* < 0.05) and fluorouracil (5-Fu; both *p* < 0.01) compared with untreated cells.

### Compound Kushen injection induces autophagy but does not alter the apoptosis of SW480 and SW620 CRC cells in vitro

**Figure 6:**
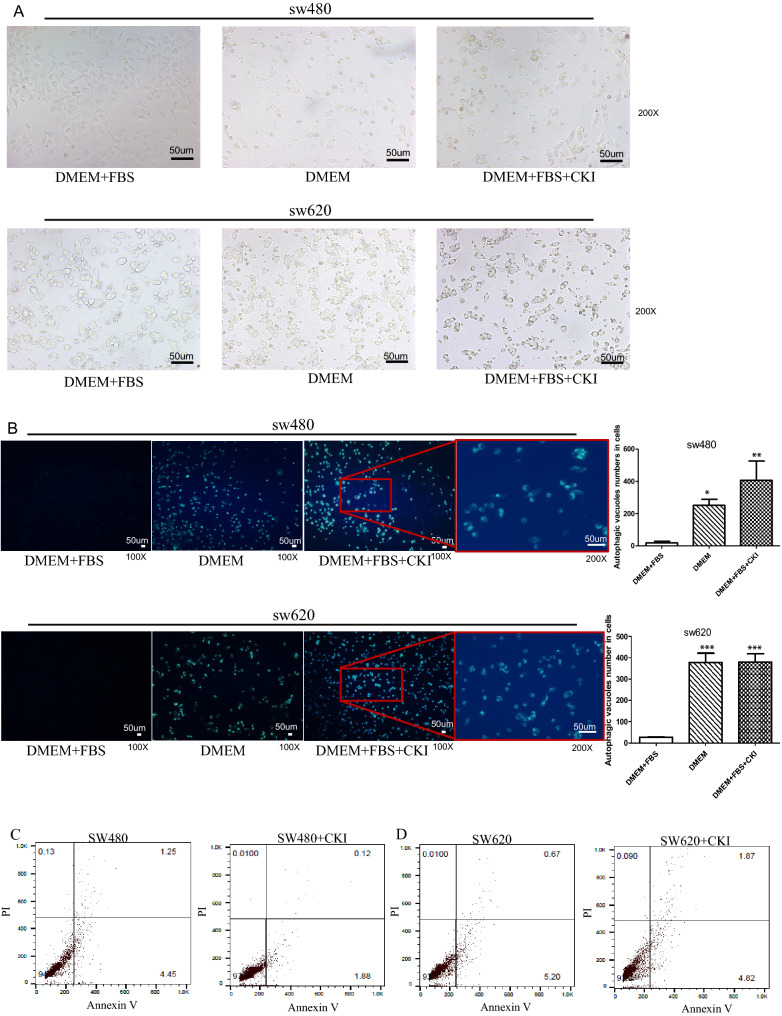
Compound Kushen injection induces autophagy but does not alter the apoptosis of SW480 and SW620 CRC cells in vitro. SW480 and SW620 cells were incubated in regular DMEM as a negative control and in serum-depleted medium as a positive control for 24 h. Both control cells and CKI-treated cells were incubated with a working solution of Autophagy Blue for 30 min at 37 °C in a 5% CO_2_ incubator and then washed four times with wash buffer. (**A**) Cells were imaged under white light under a microscope. (**B**) Cells were imaged under a fluorescence microscope with a DAPI channel. Autophagy is indicated by the bright blue dot staining of autophagic vacuoles. Each data point represents the mean ± SD from three independent experiments. ***p < 0.001, compared with negative control. The scale bar represents a length of 50 um in each of the images and the image was created using GraphPad Prism 5.01 software. (**C**,**D**) Cell apoptosis was assessed by the Annexin-V assay.

To investigate CKI-induced autophagy in SW480 and SW620 CRC cells, we measured vesicles that were labeled with Autophagy Blue™, a widely used specific autophagosome marker, to analyze the activity of autophagy. As shown in Fig. 6A,B, both SW480 and SW620 cells exhibited significant changes in morphology compared with cells that were cultured in a medium that contained serum after starvation in serum-free medium or CKI incubation for 24 h (both *p* < 0.001). Cells that were treated with CKI in the culture medium with serum exhibited obvious autophagic vacuoles, indicated by Autophagy Blue™ staining. To further determine the effect of CKI on the apoptosis of SW480 and SW620 cells, we performed apoptosis assays. As shown in Fig. 6C,D, CKI did not affect the apoptosis of SW480 cells and SW620 cells.

### Compound Kushen injection prevents the development of AOM/DSS-induced CRC in vivo

**Figure 7:**
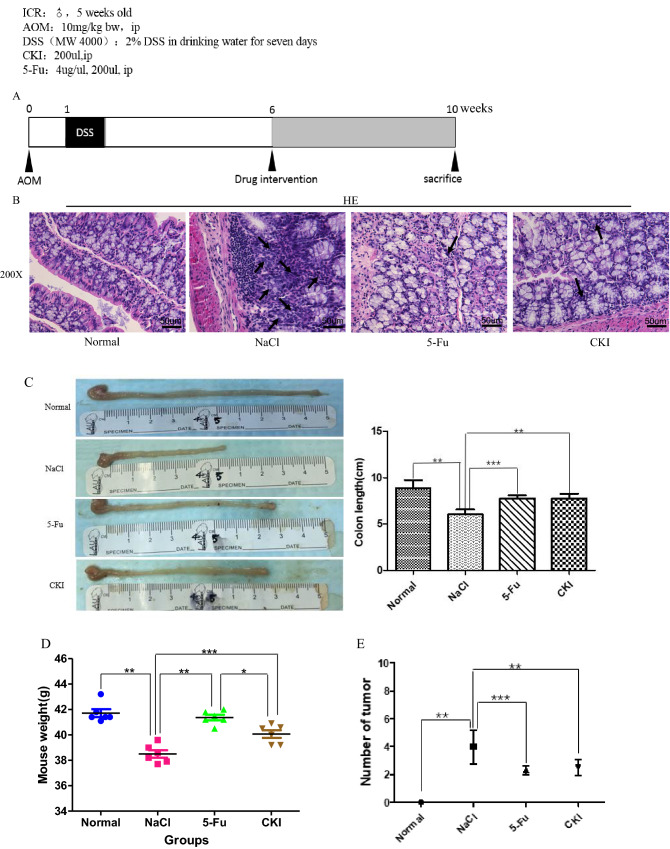
Compound Kushen injection inhibits AOM/DSS-induced colon tumor progression in mice. (**A**) Schematic drawing of Colitis Colon Cancer Model induced by AOM/DSS in ICR mice. (**B**) ICR mice were divided into a normal group and AOM/DSS group. Mice in the AOM/DSS group were given 0.9% NaCl, CKI, and 5-Fu. Shown are representative histopathological images of staining in non-tumor colon tissues, AOM/DSS-derived colon tumors tissues, and drug intervention tissues stained with hematoxylin and eosin (HE). Original magnification: 200 ×. The scale bar represents a length of 50 um in each of the images. (**C**) Colon length. (**D**) Comparison of body weights in the normal group and AOM/DSS group. (**E**) Tumor number. *p < 0.05, **p < 0.01, ***p < 0.001, compared with each group. The image was created using GraphPad Prism 5.01 software.

To investigate the preventive effect of CKI on the development of CRC and induction of the cell cycle arrest of CRC cells, we first successfully constructed the AOM/DSS-induced CRC model (Fig. 7A,B), which was histologically confirmed at the endpoint of the experiment (Fig. 7B). For comparison purposes, 5-Fu was used in this study when considering its ability to prevent CRC development and arrest the cell cycle. A time-course observation is shown in Fig. 7A. The length of the colon, which was greatly shortened in AOM/DSS mice compared with normal controls, was significantly restored by 5-Fu and CKI treatment (Fig. 7C). The weight of AOM/DSS mice was significantly reduced compared with normal controls. The weight of mice that were treated with 5-Fu and CKI was significantly higher than AOM/DSS mice but slightly lower than normal controls (Fig. 7D). The total number of tumors was significantly decreased by 5-Fu and CKI treatment compared with AOM/DSS mice (Fig. 7E). As shown in Fig. 7B, AOM/DSS mice exhibited obvious tumor lesions (0.9% NaCl group), in which the center of the large intestinal gland was larger and irregular, whereas mice in the 5-Fu and CKI groups exhibited healthy tissue morphology and had no tumor focus. Altogether, these results suggest that CKI exerted comparable anti-tumor effects and significantly inhibited the AOM/DSS-induced development of CRC.

### Compound Kushen injection downregulates the expression of targets that are related to cell cycle arrest in vitro and in vivo

The cell cycle is a common target signaling pathway of CKI against CRC, which was predicted by network pharmacology and verified by RNA-Seq (SW480 and SW620 cells). However, CKI target genes that are involved in the cell cycle are not the same (Supplementary Table S4). Cell cycle-related CKI targets, verified in SW480 cells, did not overlap with those that were predicted by network pharmacology and verified by SW620 cells. Considering the consistency of the network pharmacology prediction and RNA-Seq verification results of SW620 cells and considering that SW620 cells are more representative of CRC than SW480 cells, we selected p53, CHEK1, CCND1, CDKN2A, MDM2, and p21 as key targets of CKI-induced cell cycle arrest. Among these, p53 and CHEK1 are common CKI targets for SW620 cell verification and network pharmacology prediction. CCND1, CDKN2A, and MDM2 are specific CKI targets of network pharmacology prediction, and p21 is the specific CKI target of SW620 cells.

**Figure 8:**
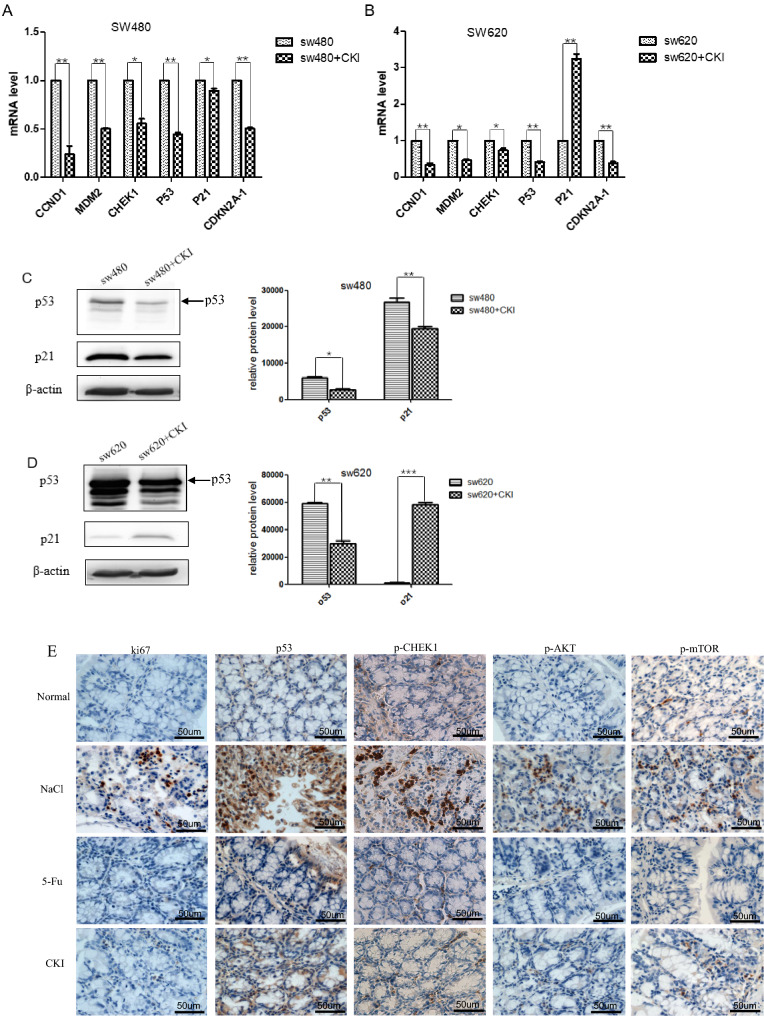

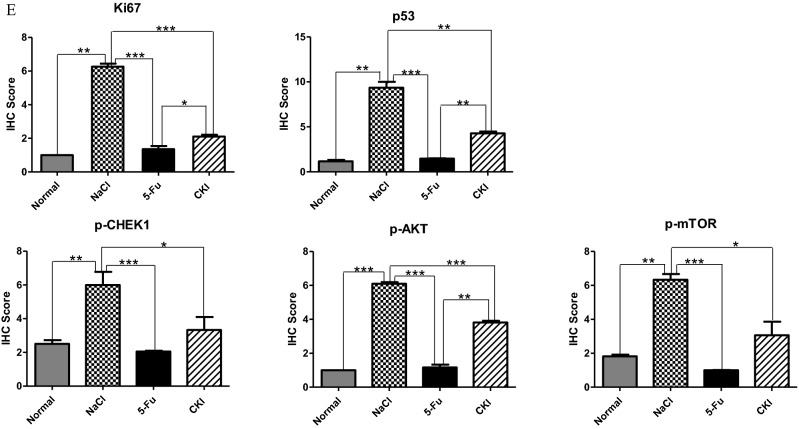
Compound Kushen injection alters the expression of targets related to cell cycle arrest in SW480 and SW620 cells. (**A**,**B**) mRNA levels of CCND1, MDM2, CHEK1, CDKN2A, p53, and p21 were analyzed by RT-qPCR in SW480 and SW620 cells. (**C**,**D**). Western blot analysis of p53 and p21 in SW480 and SW620 cells. All Western blot bands were quantified using a custom ImageJ script. The results are expressed as the mean ± standard deviation (SD) from three independent experiments. *p < 0.05, **p < 0.01, ***p < 0.001, compared with untreated group. (**E**) Immunohistochemical staining of ki67, p53, p-CHEK1, p-AKT, and p-mTOR in colorectal tissue from mice in the normal group and AOM/DSS group (400 × magnification) and total immunohistochemistry score for ki67, p53, p-CHEK1, p-AKT, and p-mTOR in colorectal tissue from mice in the normal group and AOM/DSS group. The scale bar represents a length of 50 um in each of the images. *p < 0.05, **p < 0.01, ***p < 0.001, compared with each group. The image was created using GraphPad Prism 5.01 software.

As shown in Fig. 8, the mRNA expression of *p53*, *CHEK1*, *CCND1*, *CDKN2A*, and *MDM2* was significantly downregulated in both SW480 and SW620 cells after CKI treatment. *p21* mRNA and protein expression were significantly downregulated in SW480 cells but upregulated in SW620 cells. These results confirmed the target network pharmacology prediction from the perspective of the in vitro experiments and further showed that CKI plays a pharmacological role in cell cycle arrest in CRC cells by downregulating the expression of these targets, with the exception of p21.

As shown in Fig. 2B and Supplementary Table S2, the size of the p53 node was the largest in the PPI network of CKI targets, representing the highest degree value. This indicates that p53 occupied the most important position in the entire PPI network and plays a decisive role in CKI-induced cell cycle arrest and the prevention of CRC. In the present study, we found that CKI effectively inhibited the mRNA and protein expression of mutant *p53* in SW480 and SW620 CRC cells in vitro (Fig. 8A–D) and inhibited p53 protein expression in colon tissue in the AOM/DSS-induced CRC model in vivo (Fig. 8E). We also detected the expression of ki67, phosphorylated AKT (p-AKT), p-mTOR, and p-CHEK1, which are closely related to p53, cell proliferation, and growth, by immunohistochemistry and found the high expression of ki67, mutant p53, p-CHEK1, p-AKT, and p-mTOR in colorectal tissues in the AOM/DSS group that was treated with 0.9% NaCl. After treatment with 5-Fu and CKI, the expression and degree of these five proteins in colorectal tissue in AOM/DSS mice (with 0.9% NaCl treatment) significantly decreased. We further examined expression in colorectal tissue by immunohistochemistry in the normal group and AOM/DSS group (with 0.9% NaCl treatment) (Fig. 8E). The expression of ki67, mutant p53, p-CHEK1, p-AKT, and p-mTOR positively correlated with the AOM/DSS group that was treated with 0.9% NaCl. Among these, the effect of 5-Fu was more apparent, which was close to normal tissue. Additionally, although the effect of CKI was slightly weaker than the positive control drug 5-Fu compared with the AOM/DSS group that was treated only with 0.9% NaCl, the effect of these five proteins was still significantly reduced.

Altogether, these results demonstrate that cell cycle arrest that is induced by p53 and CHEK1 is an important mechanism by which CKI prevents CRC development. We successfully constructed a “bioactive compound (genistein, hesperetin, lanceolarin, and trifolirhizin)-target (p53 and CHEK1)-cell cycle pathway” network for the anti-CRC effect of CKI.

## Discussion

As an anti-tumor TCM preparation that was approved by the Chinese Food and Drug Administration in 1995, CKI has been widely used for the clinical treatment of various malignant tumors, including CRC, in China, with a remarkable curative effect^6,9,12^. However, because of a lack of evidence from basic research, the potential mechanism of action of CKI for cancer treatment is still not understood. In recent years, the application of network pharmacology, RNA-Seq technology, and bioinformatics has become a good research tool for screening bioactive ingredients with anti-cancer properties and predicting target genes and related signaling pathways. The application of in vitro cells and in vivo animal models has also provided robust technical support for verifying the biological effects of CKI^16–27^.

Recently, several research groups have successfully constructed CKI anti-cancer “compound-protein target-pathway” networks using network pharmacology. Compound Kushen injection was shown to play an anti-cancer role through multiple components, targets, and pathways. These anti-cancer mechanisms of action of CKI have demonstrated its anti-pan-cancer activities and specificity for some cancer types^16–20^. He et al. speculated that the potential molecular mechanism of action of CKI’s anti-pan-cancer actions involves promotion of the p53- and PI3K/Akt-mediated apoptosis of tumor cells^16^. For individual cancers, Zhou et al. constructed a pharmacological “active ingredient-protein target-signaling pathway” network for gastric cancer, esophageal cancer, lung cancer, and breast cancer^17–20^. Meng et al. showed that CKI play a therapeutic role in lung cancer by regulating some important pathways, such as pathways in cancer, proteoglycans in cancer, the PI3K-Akt signaling pathway, non-small-cell lung cancer, and small-cell lung cancer^19^.

In the present study, 34 protein targets, which mapped to 82 compounds, were selected to construct the “herb-compound-protein target” network, and 33 signaling pathways were predicted to be targets of anti-CRC CKI (Fig. 3B, Supplementary Table S4). In addition to various cancer-related pathways, the predicted pathways mainly included adherens junction, transcriptional misregulation in cancer, signaling pathways regulating pluripotency of stem cells, Wnt, focal adhesion, Happo, PI3K-Akt, central carbon metabolism in cancer, cell cycle, HIF1, ErbB, FoxO, CRC, microRNAs in cancer, proteoglycans in cancer, p53, and pathways in cancer (Supplementary Table S4). The PPI analysis suggested that these predicted pathways are mainly associated with multiple hub protein targets, such as TP53, CCND1, CDKN2A, MDM2, CTNNB1, EGF, MTOR, CHEK1, and IGF1 (Fig. 2C). By integrating the findings of other research groups^17–20^ and our present findings, we found that different cancers have many common CKI target pathways (such as HIF1, PI3K-Akt, ErbB, FoxO, and p53; Supplementary Table S5). Although there may be some overlap, the hub protein targets were quite different. Therefore, the predicted hub protein targets and pathways of anti-CRC CKI were validated by primary (SW480) and metastatic (SW620) isogenic CRC cell lines^28^. mRNA expression of the *TP53*, *CHEK1*, *DNMT1*, *ANP32A*, *DHFR*, *TOP1*, *MMP1*, *DICER1* genes was confirmed by RNA-Seq, and the results were consistent with the predicted network pharmacology results. As shown in Fig. 4 and Supplementary Table S4, we enriched 23 and 32 KEGG pathways of CKI in SW480 and SW620 cells, respectively. The CKI target pathways that were enriched in SW480 cells were significantly different from those that were predicted by network pharmacology and enriched in SW620 cells. The cell cycle was the only CKI target pathway that was enriched in network pharmacology, SW480 cells, and SW620 cells. Among the CKI target pathways (except the cell cycle) enriched in SW620 cells, 16 were consistent with our network pharmacology prediction results, and most of them were also consistent with the prediction results of gastric cancer, esophageal cancer, lung cancer, and pan-cancer (Supplementary Table S5). This type of experimental sequencing verification illustrates the reliability of the network pharmacology analysis but also indicates that we need to combine differences of different tumors and heterogeneity of the same tumor to study the anti-cancer effects and mechanism of action of CKI.

As mentioned above, preliminary verification by RNA-Seq at the cell level showed that the cell cycle, p53 signaling pathway, FoxO signaling pathway, ErbB signaling pathway, Viral carcinogenesis, and proteoglycans in cancer were key signaling pathways that are targeted by CKI, and most of them are involved in cell cycle progression, proliferation, apoptosis, autophagy, and growth of CRC cells. RNA-Seq also showed that p53 and CHEK1 are key target proteins of CKI in inhibiting CRC and inducing SW620 cell cycle arrest. We also found that CKI induced the cell cycle arrest (Fig. 5A) and autophagy (Fig. 6A,B) of SW480 and SW620 cells, inhibited their proliferation in vitro (Fig. 5B), and effectively inhibited AOM/DSS-induced carcinogenesis and the development of CRC in vivo (Fig. 7).

Cell cycle control is one of the major regulatory mechanisms of cell growth, and abnormal regulation of the cell cycle is a notable characteristic of cancer cells^29,30^. In the present study, we utilized a human CRC cell model and AOM/DSS-induced mouse colorectal carcinogenesis model to validate predicted potential key markers that are associated with the cell cycle in vitro and in vivo. The present study showed that CKI significantly inhibited the growth and induced the autophagy of tumor cells. After CKI treatment, the SW480 cell cycle was significantly inhibited in the G0/G1 phase, and the SW620 cell cycle was arrested in the G2/M phase. The discrepancy in CKI-mediated cell cycle control between SW480 and SW620 cells may be attributable to differences in genetic backgrounds or signaling networks between the two cell lines. The SW480 cell line was originally established from primary adenocarcinoma of the colon. It has a G → A mutation in codon 273 of the *p53* gene. By comparison, the SW620 cell line was established from a lymph node in the same manner that the SW480 cell line was initiated from primary adenocarcinoma the previous year^31^. The quantitative real-time reverse-transcription polymerase chain reaction (qRT-PCR) results indicated that mRNA levels of *CCND1*, *CHEK1*, *p53*, *CDKN2A*, and *MDM2* were significantly downregulated in SW480 and SW620 cells, but the mRNA level of p21 was upregulated in SW620 cells and downregulated in SW480 cells. In response to various cellular signals, p53 becomes activated to function as a transcription factor that transcribes a genetic program to accomplish a number of different functions, such as activating DNA repair, inducing cell cycle arrest, and initiating autophagy^32,33^. The upregulation of p53 protein can initiate p21-dependent cellular growth arrest. P21CIPI/WAF1 protein inhibitor binds to and inhibits the activity of cyclin-CDK2 complexes, thereby functioning as a regulator of cell cycle progression. The expression of this gene is tightly controlled by the tumor suppressor protein p53^34^. Thus, the p53-p21 axis is regarded as a classic pathway for G2/M phase arrest that is induced by DNA-damaging agents^35^. However, when p53 mutates, the original function of p53 is inhibited. This was supported by the present findings in SW480 and SW620 cells, which expressed high levels of mutant p53 protein. Consistent with the qRT-PCR data, the expression levels of TP53 and p21 proteins were downregulated in SW480 cells after CKI treatment, the expression levels of TP53 were downregulated in SW620 cells, and the expression levels of p21 were upregulated in SW620 cells. One explanation for this differential effect of p21 with CKI treatment is that p53 has multiple downstream target genes, in addition to p21, following CKI treatment. We found that CKI may inhibit CRC proliferation by downregulating mutant p53 protein expression, thereby regulating cell cycle progression. Furthermore, we also investigate the effect of CKI on p21 and proliferation inhibition in p53 wt and p53 null CRC cell lines. As shown in Figure S2 and S3, the proliferation of HCT116 and HCT116 p53−/− cells was significantly inhibited following treatment with CKI. In HCT116 cells, CKI can promote the expression of p21 protein and inhibit the expression of wild-type p53 protein, while in HCT116 p53−/− cells, CKI can also promote the expression of p21, but inhibit the expression of a shorter isoform of wild-type p53. One explanation for this is that a WT TP53 isoform can also act as an oncogene and add a new layer to the already complex p53 signaling network^36–38^.

Autophagy is an evolutionarily conserved lysosomal self-digestion process that plays a dual role in tumorigenesis and cancer therapy. With regard to cancer, many links exist between autophagy and p53. In this study, we found that CKI can induce autophagy in SW480 and SW620 cells (all with mutant p53), and this autophagy behavior can be inhibited by 3-MA. These findings suggest that p53 may modulate autophagy and that p53 status could determine cell fate following anti-cancer drug-induced autophagy and help maintain autophagic homeostasis.

Based on the results of the “herb-compound-target” network (Fig. 2B), we identified one bioactive compound (from Baituling) that targets CHEK1 and three bioactive compounds (from Kushen) that target p53. These four include trifolirhizin, genistein-7-rutinoside, lanceolarin, and hesperetin-7-*O*-rutimoside. Zhang et al. found that human gastric cancer inhibition by trifolirhizin in vitro and in vivo was facilitated by autophagy, mitochondrial mediated programmed cell death, G2/M phase cell cycle arrest, and inhibition of the m-TOR/PI3K/AKT signaling pathway^39^. Sun et al. reported that trifolirhizin induced autophagy-dependent apoptosis in colon cancer via AMPK/mTOR signaling^40^. Combined with our results, trifolirhizin appears to induce CRC cell cycle arrest in the G2/M phase by targeting CHEK1. Zhang et al. and Han et al. reported that genistein promoted colon cancer cell growth inhibition and facilitated apoptosis and cell cycle arrest in the G2/M phase through an ATM/p53-p21 cross-regulatory network and the FOXO3-p53(mut) complex^41,42^. Except apoptosis, our results also showed that CKI induced SW620 cell cycle arrest in the G2/M phase, suggesting that genistein induced cell cycle arrest by targeting mutant p53. Nalini et al. demonstrated the efficiency of hesperetin against 1,2-dimethylhydrazine-induced rat colon carcinogenesis^43^. Alshatwi et al. reported that the apoptotic effect of hesperetin on human cervical cancer cells was mediated through cell cycle arrest, death receptor, and mitochondrial pathways^44^. Altogether, we suggest that the “CKI bioactive compound (trifolirhizin, genistein-7-rutinoside, lanceolarin, and hesperetin-7-*O*-rutimoside)-target (mutant p53)-pathway (CHEK1-G2/M cell cycle arrest)” network is an important mechanism by which CKI prevents CRC.

Compound Kushen injection is mainly used for the comprehensive treatment of advanced CRC. Our results in SW480 cells suggest that although its mechanism of action is different from SW620 cells, CKI could be used for the treatment of early carcinoma in situ, based on its effect of inducing cell cycle arrest. 5-Fluorouracil is frequently used for CRC treatment because of its ability to induce CRC cell cycle arrest^45^. Thus, 5-Fu was used as a positive control drug in the present study. Both the in vitro and in vivo studies showed that CKI exerted similar anti-CRC effects as 5-FU, thus providing a good experimental basis for the clinical use of CKI alone for the treatment of CRC. Additionally, mutant p53 is overexpressed in patients with CRC and promotes tumor growth in vivo. Therefore, the inhibition of mutant p53 may be a useful strategy for the treatment of mutant p53-overexpressing CRC in patients.

In conclusion, based on network pharmacology analyses, the active components of CKI for the treatment of CRC and their corresponding targets and pathways were screened. Our results were verified both in vivo and in vitro using advanced molecular biology and RNA-Seq approaches. Our data demonstrate potential pharmacological mechanisms of action of CKI. Considering the diversity of effective components, targets, and pathways of CKI, further research and validation are needed to clarify its anti-CRC activity.

## Materials and methods

### Active ingredients in CKI

The ingredients of Kushen and Baituling were obtained from the Traditional Chinese Medicine Systems Pharmacology (TCMSP) database (http://tcmspw.com/tcmsp.php)^46^, the Traditional Chinese Medicine Information (TCMID) database (http://119.3.41.228:8000/tcmid/search/)^47^, and the literature. To screen components with high biological activity in the human body, we employed an important ADME (Absorption, Distribution, Metabolism, Excretion)-related property of injection, Drug-Likeness (DL), to explore the potential bioactive compounds in CKI^48^. Ingredients with DL < 0.18 were removed from the study.

### Potential targets of CKI

To identify potential targets of CKI, one drug target prediction using the PharmMapper database (http://lilab.ecust.edu.cn/pharmmapper/) was used. It was designed to identify potential targets for small molecules using a reverse pharmacophore mapping approach^49^. Structural information for the compounds was first obtained from the National Center for Biotechnology Information PubChem database (https://pubchem.ncbi.nlm.nih.gov/). An SDF file of compounds was then uploaded into PharmMapper database, and the Human Protein Targets Only database was selected^50^. The top 30 potential targets for each compound were obtained and sorted by the normalized fit score. Official genes names were taken from UniProt (http://www.uniprot.org/) by confining the species to “*Homo sapiens*”.

### Colorectal cancer-related targets

Colorectal cancer-related target genes were identified in four databases using “colorectal cancer” as keywords, and the species was set to *Homo sapiens*. We used the TTD (https://db.idrblab.org/ttd/), OMIM (https://omim.org/)^51^, GeneCards (https://www.genecards.org)^52^, and DisGeNET (https://www.disgenet.org/home/)^53^ databases. The search results in the DisGeNet database are sorted according to the disease specificity index (DSI), and the targets with DSI below median which had less relevant to colorectal cancer were discarded. The search results of the GeneCards database are sorted by “Relevance score” and the top 200 targets are obtained. All targets retrieved by TTD and OMIM are included in the study. Disease-related genes were compared with potential targets for the active components, providing potential targets of anti-cancer components of CKI.

### Protein–protein interaction data

We obtained PPI data from the Search Tool of the Retrieval of Interacting Genes (STRING) database (https://string-db.org/), with the species limited to “*Homo sapiens*”^54^. Simultaneously, the PPI interactive network was constructed and visualized using Cytoscape 3.7.1 (http://www.cytoscape.org/) software. The Cytohubba plug-in was used to select center targets of CKI for CRC.

### Construction of herb-compound-target network

Cytoscape 3.7.1 (http://www.cytoscape.org/)^55^ was used to construct a network that would facilitate the scientific interpretation of complicated relationships among herbs, compounds, and targets. Such parameters as Characteristic Path Length and Average Number of Neighbors were calculated using NetworkAnalyzer^56^.

### Gene ontology and KEGG pathway enrichment analyses

Target genes were used for GO (Gene ontology) term and KEGG (Kyoto Encyclopedia of Genes and Genomes) pathway enrichment analyses using DAVID (http://david.abcc.ncifcrf.gov/)^57^. Results with *p* < 0.05 were considered statistically significant^58^.

### Cell lines and cell culture

The SW480 and SW620 cell lines were purchased from the Cell Bank of Chinese Academy of Sciences (Shanghai, China) and cultured in Dulbecco’s Modified Eagle Medium (DMEM) supplemented with 10% fetal bovine serum, 1% penicillin, and 1% streptomycin.

### RNA extraction and sequencing

Cells were cultured in six-well plates at a seeding density of 5 × 10^5^ cells/well and treated with CKI (2 mg/ml, based on the total alkaloid concentration in CKI^59^) for 24 h. Total RNA was isolated using the mirVana PARIS Kit (Thermo Fisher Scientific) according to the manufacturer’s protocol. Gene expression profiles were experimentally analyzed using the Illumina HiSeq 2000 RNA Sequencing platform. This dataset showed gene-level transcription estimations (log2[x + 1] transformed RSEM normalized count). Genes were mapped onto human genome coordinates using HUGO probe Map.

### Flow cytometry assays

For cell cycle analysis, SW480 and SW620 cells (1 × 10^6^ cells/well) were seeded into six-well plates, cultured in complete culture medium overnight, and then exposed to CKI (2 mg/ml, based on the total alkaloid concentration in CKI) for 24 h. Nuclear DNA was analyzed using the BD Cycletest Plus DNA Kit (BD Biosciences, San Jose, CA, USA) according to the manufacturer’s instructions. Apoptosis assays were performed immediately after the cells were cultured for 24 h and counted at a density of 1 × 10^6^ cells/ml. Apoptosis was monitored using the Annexin V-FITC Apoptosis Detection Kit (Beyotime, Shanghai, China). Cells that stained negative for both propidium iodide and annexin V were considered viable cells. Propidium iodide-negative and annexin V-positive stained cells were considered early apoptotic cells. Propidium iodide-positive and annexin V-positive stained cells were considered to be in later stages of apoptosis. Stained cells were detected and quantified using a fluorescence-activated cell sorting (FACS) flow cytometer (BD Biosciences, San Jose, CA, USA). All FACS results were analyzed using FlowJo 7.6 software.

### Cell viability assays

The proliferation of SW480 and SW620 cells was assessed using the Cell Counting Kit-8 (Dojindo, Rockville, MD, USA) according to the manufacturer’s instructions. Cells were seeded in 96-well plates at a density of 1 × 10^5^ cells/ml per well and then exposed to DMEM, 5-Fu (10 µg/ml), or CKI for 24 or 48 h. Compound Kushen injection was used (Zhendong, Changzhi, Shanxi, China) at a final concentration of 2 mg/ml (based on the total alkaloid concentration in CKI). The samples run in triplicate in the Cell Viability experiments. Optical densities were obtained by reading plates at 450 nm using a 96-well micro test spectrophotometer (Bio-Rad, Hercules, CA, USA).

### Autophagy assays

Autophagy was analyzed using the Cell Meter Autophagy assay. SW480 and SW620 cells were seeded in 96-well plates at a density of 2 × 10^4^ cells/ml in 100 ul DMSO overnight. A non-induced negative control cell population at the same density as the induced population was cultured for every labeling condition. The medium was then removed, and 100 µl of Autophagy Blue working solution (AAT Bioquest, Sunnyvale, CA, USA) was added to each well. The samples run in triplicate and were then incubated at 37 °C in a 5% CO_2_ incubator for 1 h. After three washes, fluorescent intensity was monitored with a fluorescence microscope using the DAPI channel.

### Animal model experiments

All animal care and handling guidelines were approved by the Animal Care Committee of Peking University People’s Hospital. Time-course observations of AOM/DSS-induced colorectal carcinogenesis were conducted in mice^60^. Five-week-old male ICR mice were kept under specific-pathogen-free conditions and received a single intraperitoneal injection of AOM (10 mg/kg body weight; Sigma-Aldrich, St. Louis, MO, USA), followed by 1 week of 2% DSS supplementation (MP Biomedicals, Irvine, CA, USA) in drinking water, after which the mice were maintained on regular water for 28 days. The mice in the AOM-DSS group were then randomly divided into three subgroups (*n* = 6/group). At week 7, these three groups of mice were injected intraperitoneally with 200 µl of 0.9% NaCl (original injection), 200 µl of 5-Fu (4 mg/ml), or 200 µl of CKI (original injection). At approximately 4 weeks after the drug intervention, the mice were sacrificed, and colorectal tissues were sectioned for histological analysis.

### Validation of results by real-time qRT-PCR

SW480 and SW620 cells were incubated at 37 °C in a humidified incubator after treatment with CKI (2 mg/ml, based on the total alkaloid concentration in CKI) for 24 h. At the same time, phosphate-buffered saline (PBS) was used as a control. Total RNA was extracted using an RNeasy Mini kit (Qiagen, Hilden, Germany) according to the manufacturer’s instructions, and cDNA was synthesized with a SuperScript III Synthesis Reagent Kit (Invitrogen, Carlsbad, CA, USA). qRT-PCR was performed as described previously^61^. The primers are listed in the Supplementary Materials (Supplementary Table S6). Amplification was conducted and analyzed using a CFX96 analyzer (Bio-Rad, Hercules, CA, USA).

### Western blot analysis

Western blot and immunoprecipitation were performed as described previously^61^. The cells of the two colorectal cancer cell lines, SW480 and SW620, were collected in the logarithmic growth phase, and the cell concentration was adjusted to 5 × 10^5^ cells/ml. CKI (2 mg/ml, based on the total alkaloid concentration in CKI) was added to each cell culture, and cells were incubated for 24 h. The cells were then collected, washed twice with cold PBS, and harvested in 500 ml cell lysis buffer (containing incubation buffer, 20% Triton X-100, protease inhibitor, sodium fluoride, and sodium orthovanadate solution) for 30 min on ice, briefly mixed, and centrifuged at 14,000 × *g* at 4 °C for 30 min. The total protein concentration was determined by a BCA Protein Assay Kit (Thermo Scientific Pierce, Waltham, MA, USA). Equal amounts of cellular proteins were subjected to 8% sodium dodecyl sulfate–polyacrylamide gel electrophoresis and then transferred to polyvinylidene difluoride membranes. The membranes were blocked with Tris-buffered saline (TBS) buffer containing 5% skim milk powder at room temperature for 1 h, and then incubated with primary antibodies overnight at 4 °C. The blots were washed three times in TBS with Triton X-100 and incubated with horse radish peroxidase-conjugated secondary antibody (1:1000; ZSGB Biotechnology, Beijing, China) for 1 h at room temperature. Blots were then visualized with enhanced chemiluminescence reagent (Thermo Fisher Scientific, Waltham, MA, USA). All grey values of the protein bands were performed using custom ImageJ (National Institutes of Health; http://rsb.info.nih.gov/ij/) script.

The antibodies are listed in the Supplementary Materials (Supplementary Table S7).

### Immunohistochemistry

Colorectal tissues from AOM/DSS mice were deparaffinized in xylene and then rehydrated in a decreasing series of ethanol. The specimens were retrieved by a water bath at 95℃ for 15 min in 0.01 M citrate buffer (pH 6.0), and then endogenous peroxidase was deactivated by incubating with 3% hydrogen peroxide for 15 min, after which the sections were washed with phosphate-buffered saline (PBS). Nonspecific binding was blocked by incubating with 10% normal goat serum (ZSGB, Beijing, China) for 60 min. The sections were then incubated with a working solution of p53, p-CHEK1, ki67, p-AKT, or p-mTOR primary antibody. After washing with PBS, the sections were incubated with horseradish peroxidase-conjugated rabbit anti-goat immunoglobulin G and stained with 3,3′-diaminobenzidine and then counterstained with hematoxylin. As negative controls, PBS was used instead of the primary antibody. Digital images were acquired using a Zeiss AXIO Scope A1 digital camera microscope (Zeiss, Jena**,** Germany). The antibodies and dyes are listed in the Supplement Materials (Supplementary Table S7). The immunohistochemistry staining intensity and extent of the staining area were classified as the following^62^: 1 (no or weak staining), 2 (moderate staining), and 3 (strong staining). The percentage of positive cells was defined as the following: 0 (0%), 1 (1–24%), 2 (25–49%), 3 (50–74%), 4 (75–100%). The final immunoreactive score (0 to 12) was determined by multiplying the intensity score by the percentage of staining score.

### Statistical analysis

The statistical analysis was performed using GraphPad Prism 5.01 software. All of the experiments were repeated at least three times. ANOVA analysis and Student’s *t*-test were used to evaluate the data of experiments. Two-tailed values of *p* < 0.05 were considered statistically significant.

### Approval for animal experiments

The animal experiments were approved by the Ethics Committee of Peking University People’s Hospital. All animal care and handling procedures were performed according to the National Institutes of Health's Guide for the Care and Use of Laboratory Animals. The study was performed in compliance with ARRIVE 2.0 guidelines (https://arriveguidelines.org).

## Supplementary Information

Supplementary Information.
