# Supplementary material for: Cell cycle arrest is an important mechanism of action of compound Kushen injection in the prevention of colorectal cancer
Source: Sci Rep. 2022 Mar 14;12:4384. doi: 10.1038/s41598-022-08336-4 (PMC8921286; doi:10.1038/s41598-022-08336-4)
Supplement: Supplementary file 1 — Supplementary Information. [file 41598_2022_8336_MOESM1_ESM.docx]

**Cell cycle arrest is an important mechanism of action of compound Kushen injection in the prevention of colorectal cancer**
Jie Sun^1,+^, Mei Li^1,+^, Tingru Lin^1,2+^, Di Wang^1^, Jingyi Chen^2^, Yu Zhang^2^, Qing Mu^1^, Huiting Su^1^, Na Wu^1^, Aiyu Liu^1^, Yimeng Yu^1^, Yulan Liu^2^, Shaojie Wang^3^, Xin Yu^4^, Jingzhu Guo^5,*^, Weidong Yu^1,*^

·Authors’ affiliations:
^1^Department of Central Laboratory & Institute of Clinical Molecular Biology, Peking University People’s Hospital, Beijing, China
^2^Department of Gastroenterology, Peking University People’s Hospital, Beijing, China
^3^Department of Traditional Chinese Medicine, Peking University People’s Hospital, Beijing, China

^4^Department of hepatobiliary Surgery, Peking University People’s Hospital, Beijing, China,
^5^Department of Pediatric, Peking University People’s Hospital, Beijing, China
*Corresponding authors:
Jingzhu Guo, [guojingzhu@pkuph.edu.cn](mailto:guojingzhu@pkuph.edu.cn)
Weidong Yu, [weidongyu@bjmu.edu.cn](mailto:weidongyu@bjmu.edu.cn)
+Jie Sun, Mei Li and Tingru Lin contributed equally to this study

Supplementary Table S1 A list of target genes and corresponding compounds in the treatment of colorectal cancer with CKI.

| PubChem CID | Compound | Herb | Target | Uniprot ID |
| --- | --- | --- | --- | --- |
| 12442899 | Sophoranol | Kushen | IRF1 | P10914 |
| 509245 | sophoraflavanone B | Kushen | IL10 | P22301 |
| 259846 | lupeol | Kushen | MDM2 | Q00987 |
|  |  |  | MTOR | P42345 |
| 115012 | soyasapogenol B | Kushen | MDM2 | Q00987 |
|  |  |  | MTOR | P42345 |
|  |  |  | ANP32A | P39687 |
| 3082765 | dehydromiltirone | Kushen | MDM2 | Q00987 |
|  |  |  | ANP32A | P39687 |
|  |  |  | EGF | P01133 |
|  |  |  | MTOR | P42345 |
| 102073915 | kusulactone | Kushen | MDM2 | Q00987 |
|  |  |  | MTOR | P42345 |
|  |  |  | ANP32A | P39687 |
| 91572 | Phaseolin | Kushen | MDM2 | Q00987 |
|  |  |  | MTOR | P42345 |
|  |  |  | EGF | P01133 |
|  |  |  | ANP32A | P39687 |
| 91510 | maackiain | Kushen | ERBB2 | P04626 |
| 10854625 | kushenol S | Kushen | ERBB2 | P04626 |
| 5319307 | matsukaze lactone | Kushen | ERBB2 | P04626 |
| 5281611 | Resokaempferol | Kushen | HOXB13 | Q92826 |
|  |  |  | MAP2K2 | P36507 |
| 42607818 | sophoranochromene | Kushen | HOXB13 | Q92826 |
| 91466 | matrine | Kushen | UNG | P13051 |
|  |  |  | PTPRJ | Q12913 |
| 10659287 | (-)-14β-hydroxymatrine | Kushen | UNG | P13051 |
|  |  |  | PTPRJ | Q12913 |
| 114850 | Oxymatrine | Kushen | UNG | P13051 |
|  |  |  | PTPRJ | Q12913 |
| 165549 | sophoridine | Kushen | UNG | P13051 |
|  |  |  | PTPRJ | Q12913 |
| 5271984 | isomatrine | Kushen | UNG | P13051 |
|  |  |  | PTPRJ | Q12913 |
| 115269 | sophocarpine | Kushen | UNG | P13051 |
|  |  |  | PTPRJ | Q12913 |
| 24721085 | Oxysophocarpine | Kushen | UNG | P13051 |
|  |  |  | PTPRJ | Q12913 |
| 126455735 | (-)-leontalbinine N-oxide | Kushen | UNG | P13051 |
|  |  |  | PTPRJ | Q12913 |
| 169014 | sophoramine | Kushen | UNG | P13051 |
|  |  |  | PTPRJ | Q12913 |
| 3041752 | lehmannine | Kushen | UNG | P13051 |
|  |  |  | PTPRJ | Q12913 |
| 10742453 | kushenol P | Kushen | UNG | P13051 |
|  |  |  | MPO | P05164 |
| 10835998 | kushenol R | Kushen | UNG | P13051 |
| 10598514 | kushenol T | Kushen | UNG | P13051 |
| 44563198 | kurarinol | Kushen | UNG | P13051 |
| 72936 | (-)-sophoraflavanone G | Kushen | UNG | P13051 |
| 10599228 | kushenol X | Kushen | UNG | P13051 |
| 381851 | kushenol N | Kushen | UNG | P13051 |
| 133561937 | 2'-methoxykurarinone | Kushen | UNG | P13051 |
| 5275227 | leachianone G | Kushen | UNG | P13051 |
| 20056309 | sophoraflavoside Ⅰ | Kushen | UNG | P13051 |
| 68406 | Octacosanol | Kushen | UNG | P13051 |
| 14104288 | 3,7,4'-Trihydroxy-5-methoxy-8-prenylflavanone | Kushen | UNG | P13051 |
| 15953774 | 2-(2,4-Dihydroxy-5-prenylphenyl)-5,6-methylenedioxybenzofuran | Kushen | UNG | P13051 |
| 129716399 | 8-Isopentenyl-kaempferol | Kushen | UNG | P13051 |
| 44584550 | koumidine | Kushen | UNG | P13051 |
| 101297615 | normacusine b | Kushen | UNG | P13051 |
| 442822 | sophoraisoflavanone a | Kushen | UNG | P13051 |
| 15385683 | (+)-14α-hydroxymatrine | Kushen | UNG | P13051 |
|  |  | Kushen | PTPRJ | Q12913 |
| 14379237 | 5,6-dehydrolupanine | Kushen | MTOR | P42345 |
|  |  |  | EGF | P01133 |
|  |  |  | ANP32A | P39687 |
| 6326060 | sophojaponicin | Kushen | MTOR | P42345 |
|  |  |  | DAB2 | P98082 |
| 44257277 | genistein-7-O-rutinoside | Kushen | TP53 | P04637 |
| 5492234 | lanceolarin | Kushen | TP53 | P04637 |
| 623329 | (-)-12-ethylsophoramine | Kushen | EGF | P01133 |
|  |  |  | ANP32A | P39687 |
|  |  |  | PTPRJ | Q12913 |
| 3085182 | Manmanine | Kushen | EGF | P01133 |
|  |  |  | ANP32A | P39687 |
| 14353465 | 4'-hydroxyisolonchocarpin | Kushen | EGF | P01133 |
| 162147 | Aloperine | Kushen | EGF | P01133 |
|  |  |  | ANP32A | P39687 |
| 7014 | sparteine | Kushen | EGF | P01133 |
|  |  |  | ANP32A | P39687 |
| 71317062 | Genistein-7-O-glucoside | Kushen | TYMS | P04818 |
|  |  |  | MAP2K2 | P36507 |
| 10496772 | kushecarpin C | Kushen | PTPRJ | Q12913 |
| 73145 | β-Amyrin | Kushen | ANP32A | P39687 |
| 442827 | trifolirhizin | Kushen | AURKA | O14965 |
|  |  |  | DHFR | P00374 |
|  |  |  | CHEK1 | O14757 |
|  |  |  | IGF1 | P05019 |
| 44257441 | (-)-Maackiain-3-O-glucoside | Kushen | DAB2 | P98082 |
| 14841223 | Trifolirhizin 6'-O-malonate | Kushen | DAB2 | P98082 |
| 10982109 | Specionin | Kushen | BIRC5 | O15392 |
| 21721878 | kushenol L | Kushen | PLA2G2A | P14555 |
|  |  |  | TP73 | O15350 |
|  |  |  | AXIN1 | O15169 |
| 6440079 | Hexadecyl-ferulate | Kushen | PLA2G2A | P14555 |
| 15385684 | (+)-9α-hydroxymatrine | Kushen | ALK | Q9UM73 |
|  |  |  | CCND1 | P24385 |
| 10495761 | kushecarpin A | Kushen | TP73 | O15350 |
| 14274649 | 5,9-dihydroxymatrine | Kushen | CTNNB1 | P35222 |
| 10382239 | isostrychnine | Kushen | AR | P10275 |
| 5319322 | medicagol | Kushen | TOP1 | P11387 |
| 101667015 | epilamprolobine N-oxide | Kushen | TOP1 | P11387 |
| 9576780 | macrozamin | Baituling | ERBB4 | Q15303 |
| 3220 | Emodin | Baituling | ERBB4 | Q15303 |
| 119422 | Hesperetin 7-O-rutinoside | Baituling | DICER1 | Q9UPY3 |
| 10168 | 1,8-Dihydroxy-3-carboxyanthraquinone | Baituling | DLC1 | Q96QB1 |
| 346868 | anthemisol | Baituling | CDKN2A | Q8N726 |
| 24836924 | amylum | Baituling | MMP1 | P03956 |
| 316844 | isoastilbin | Baituling | DNMT1 | P26358 |
| 101937309 | isoengelitin | Baituling | DNMT1 | P26358 |
|  |  |  | UNG | P13051 |
| 73642 | Resveratrol-3-O-β-D-glucopyranoside | Baituling | DNMT1 | P26358 |
| 12408 | Octacosane | Baituling | MDM2 | Q00987 |
| 69244670 | 3,3',5,5'-tetrahydroxy-4-methoxystilbene | Baituling | ERBB2 | P04626 |
| 128735 | Isobaimuxinol | Baituling | ERBB2 | P04626 |
|  |  |  | PTPRJ | Q12913 |
| 114829 | Liquiritigenin | Baituling | HOXB13 | Q92826 |
| 119258 | astilbin | Baituling | HOXB13 | Q92826 |
| 119422 | Hesperetin 7-O-rutinoside | Baituling | TP53 | P04637 |
| 91439 | smilagenin | Baituling | PTPRJ | Q12913 |
| 99474 | diosgenin | Baituling | PTPRJ | Q12913 |

Supplementary Table S2 Key target protein and degree value of CKI for colorectal cancer

| Target name | Degree | Target name | Degree |
| --- | --- | --- | --- |
| TP53 | 27 | MTOR | 15 |
| CCND1 | 24 | IGF1 | 14 |
| CDKN2A | 20 | CHEK1 | 13 |
| CTNNB1 | 19 | AR | 13 |
| ERBB2 | 19 | TYMS | 12 |
| MDM2 | 18 | AURKA | 12 |
| EGF | 18 | DNMT1 | 12 |

Supplementary Table S3 CKI herb-compounds-candidate targets network parameters

| Network parameters | Values |
| --- | --- |
| Numbers of nodes | 118 |
| Network density | 0.030 |
| Network diameter | 8 |
| Network heterogeneity | 1.880 |
| Average number of neighbors | 3.458 |
| Characteristic path length | 3.259 |
| Shortest paths  Network centralization | 13806(100%)  0.526 |

Supplementary Table S4 The KEGG pathway predicted by RNA-seq validation and network pharmacology

| No. | CKI Target pathways | Prediction | RNA-Seq validation | |
| --- | --- | --- | --- | --- |
|  |  | CRC (33) | SW620(32) | SW480(23) |
| 1 | cell cycle | √ (19) | √ (1) | √ (2) |
| 2 | Acute myeloid leukemia | √ (30) | √ (16) |  |
| 3 | *Chronic myeloid leukemia | √ (15) | √ (3) |  |
| 4 | *Colorectal cancer | √ (12) | √ (4) |  |
| 5 | *Endometrial cancer | √ (5) | √ (20) |  |
| 6 | *ErbB signaling pathway | √ (17)) | √ (29) |  |
| 7 | *FoxO signaling pathway | √ (14) | √ (7) |  |
| 8 | *Glioma | √ (4) | √ (17) |  |
| 9 | HTLV-1 infection | √ (27) | √ (8) |  |
| 10 | *Melanoma | √ (8) | √ (15) |  |
| 11 | *Non-small cell lung cancer | √ (6) | √ (28) |  |
| 12 | *p53 signaling pathway | √ (7) | √ (6) |  |
| 13 | *Pancreatic cancer | √ (13) | √ (18) |  |
| 14 | *pathway in cancer | √ (3) | √ (22) |  |
| 15 | *Proteoglycans in cancer | √ (9) | √ (2) |  |
| 16 | *Prostate cancer | √ (1) | √ (23) |  |
| 17 | *Viral carcinogenesis | √ (23) | √ (9) |  |
| 18 | Basal transcription factors |  | √ (32) | √ (7) |
| 19 | Homologous recombination |  | √ (12) | √ (10) |
| 20 | mRNA surveillance pathway |  | √ (25) | √ (5) |
| 21 | Nucleotide excision repair |  | √ (26) | √ (8) |
| 22 | splicsome |  | √ (14) | √ (1) |
| 23 | Adherens junction | √ (33) |  |  |
| 24 | Basal cell carcinoma | √ (28) |  |  |
| 25 | Bladder cancer | √ (2) |  |  |
| 26 | Central carbon metabolism in cancer | √ (20) |  |  |
| 27 | Focal adhesion | √ (24) |  |  |
| 28 | Happo signaling pathways | √ (22) |  |  |
| 29 | Hepatitis B | √ (29) |  |  |
| 30 | HIF1 signaling pathway | √ (18) |  |  |
| 31 | microRNAs in cancer | √ (10) |  |  |
| 32 | PI3K-Akt signaling pathways | √ (21) |  |  |
| 33 | Prolactin signaling pathway | √ (32) |  |  |
| 34 | Signaling pathways regulating pluripotency of stem cells | √ (26) |  |  |
| 35 | Thyroid cancer | √ (16) |  |  |
| 36 | Thyroid hormone signaling pathway | √ (11) |  |  |
| 37 | transcriptional misregulation in cancer | √ (31) |  |  |
| 38 | Wnt signaling pathways | √ (25) |  |  |
| 39 | Axon guicance |  | √ (21) |  |
| 40 | Epstein-Barr virus infection |  | √ (30) |  |
| 41 | Estrogen signaling pathway |  | √ (5) |  |
| 42 | Insulin signaling pathway |  | √ (27) |  |
| 43 | Lysine degradation |  | √ (31) |  |
| 44 | Mismatch repair |  | √ (13) |  |
| 45 | Propanoate metablism |  | √ (11) |  |
| 46 | Renal cell carcinoma |  | √ (19) |  |
| 47 | small cell lung cancer |  | √ (10) |  |
| 48 | Ubiquitin mediated proteolysis |  | √ (24) |  |
| 49 | Amino sugar and nucleotide sugar metabolism |  |  | √ (16) |
| 50 | ABC transporters |  |  | √ (15) |
| 51 | DNA replication |  |  | √ (12) |
| 52 | Fanconi anemia pathway |  |  | √ (11) |
| 53 | Galactose metabolism |  |  | √ (22) |
| 54 | Herpes simplex infection |  |  | √ (17) |
| 55 | Influenza A |  |  | √ (20) |
| 56 | Leishmaniasis |  |  | √ (23) |
| 57 | Ovarian sterodogenesis |  |  | √ (18) |
| 58 | pertussis |  |  | √ (14) |
| 59 | Pyrimidine metabolism |  |  | √ (9) |
| 60 | Ribosome biogenesis in eukaryotes |  |  | √ (6) |
| 61 | RIG-like receptor signaling pathway |  |  | √ (13) |
| 62 | RNA transport |  |  | √ (3) |
| 63 | Toll-like receptor signaling pathway |  |  | √ (19) |
| 64 | Tuberculosis |  |  | √ (21) |
| 65 | Valine, leucine and isoleucine degradation |  |  | √ (4) |

(number) represents the sequence number of signal pathways with statistical significance

Supplementary Table S5 The mainly KEGG pathway predicted of CKI anti-cancer by network pharmacology

| No. | CKI targeted signal pathways | Pan-C | CRC | GC | EsC | LC |
| --- | --- | --- | --- | --- | --- | --- |
|  |  | ref. 16 | Our data | ref.20 | ref.17 | ref.19 |
| 1 | Bladder cancer | √ (19) | √ (2) | √ (56) | √ (28) | √ (4) |
| 2 | HIF1 signaling pathway | √ (17) | √ (18) | √ (15) | √ (21) | √ (20) |
| 3 | PI3K-Akt signaling pathways | √ (1) | √ (21) | √ (6) | √ (1) | √ (17) |
| 4 | *Prostate cancer | √ (6) | √ (1) | √ (2) | √ (5) | √ (6) |
| 5 | *Colorectal cancer | √ (15) | √ (12) | √ (17) | √ (24) |  |
| 6 | *Chronic myeloid leukemia |  | √ (15) | √ (13) | √ (20) | √ (3) |
| 7 | *Endometrial cancer |  | √ (5) | √ (11) | √ (26) | √ (8) |
| 8 | *ErbB signaling pathway |  | √ (17) | √ (10) | √ (8) | √ (22) |
| 9 | Focal adhesion |  | √ (24) | √ (60) | √ (22) | √ (16) |
| 10 | *FoxO signaling pathway |  | √ (14) | √ (5) | √ (2) | √ (19) |
| 11 | *Glioma |  | √ (4) | √ (4) | √ (4) | √ (7) |
| 12 | *Melanoma |  | √ (8) | √ (9) | √ (18) | √ (5) |
| 13 | *Non-small cell lung cancer |  | √ (6) | √ (7) | √ (7) | √ (1) |
| 14 | *Pancreatic cancer |  | √ (13) | √ (8) | √ (19) | √ (9) |
| 15 | Hepatitis B | √ (7) | √ (29) | √ (1) |  | √ (13) |
| 16 | *p53 signaling pathway | √ (18) | √ (7) | √ (57) |  | √ (18) |
| 17 | *pathway in cancer |  | √ (3) | √ (3) |  | √ (11) |
| 18 | *Proteoglycans in cancer | √ (2) | √ (9) |  | √ (6) | √ (10) |
| 19 | Thyroid hormone signaling pathway | √ (9) | √ (11) | √ (50) |  |  |
| 20 | microRNAs in cancer | √ (3) | √ (10) |  | √ (3) |  |
| 21 | *Viral carcinogenesis |  | √ (23) | √ (58) | √ (23) |  |

Pan-C: Pan-Cancer; CRC: Colorectal Cancer; GC: Gastric Cancer; EsC: Esophageal Cancer; LC: Lung Cancer; (number) represents the sequence number of signal pathways with statistical significance

Supplementary Table S6 Sequences of primers for RT-qPCR analyses

| Primer Name | Primer Sequence (5’-3’) |
| --- | --- |
| 𝛽-actin Forward primer | 5’-CATGTACGTTGCTATCCAGGC-3’ |
| 𝛽-actin Reverse primer | 5’-CTCCTTAATGTCACGCACGAT-3’ |
| P53 Forward primer | 5’-CAAGCAATGGATGATTTGATGCT-3’ |
| P53 Reverse primer | 5’-TGGGTCTTCAGTGAACCATTGT-3’ |
| P21 Forward primer | 5’-TGTCCGTCAGAACCCATGC-3’ |
| P21 Reverse primer | 5’-AAAGTCGAAGTTCCATCGCTC-3’ |
| CCND1 Forward primer | 5’-GCTGCGAAGTGGAAACCATC-3’ |
| CCND1 Reverse primer | 5’-CCTCCTTCTGCACACATTTGAA-3’ |
| CDKN2A Forward primer | 5’-GATCCAGGTGGGTAGAAGGTC-3’ |
| CDKN2A Reverse primer | 5’-CCCCTGCAAACTTCGTCCT-3’ |
| CHEK1 Forward primer | 5’-AATTGCCATGGGACCAACC-3’ |
| CHEK1 Reverse primer | 5’-CTAGAGGAGCAGAATCGATT-3’ |
| MDM2 Forward primer | 5’-GAATCATCGGACTCAGGTACATC-3’ |
| MDM2 Reverse primer | 5’-TCTGTCTCACTAATTGCTCTCCT-3’ |

Supplementary Table S7 Antibodies or dyes used in this study

| Name | Article number | Corporation name | Country | Dilution ratio |
| --- | --- | --- | --- | --- |
| 𝛽-actin | SAB2100037 | Sigma-Aldrich | USA | 1:3000 (WB) |
| P53 | 21891-1-AP | Proteintech Group | USA | 1:1000 (WB)  1:400 (IHC) |
| P21 | #2947 | Cell Signaling Technology | USA | 1:1000 (WB) |
| Ki67 | Ab15580 | Abcam | UK | 1:200 (IHC) |
| p-AKT | #4060 | Cell Signaling Technology | USA | 1:200 (IHC) |
| p-mTOR  p-CHEK1 | #5536  #2348 | Cell Signaling Technology  Cell Signaling Technology | USA  USA | 1:200 (IHC)  1:100 (IHC) |
| HRP-labeled Rabbit Anti-Goat IgG | ZB-2306 | Zhongshan Golden-bridge Biotechnology | China | 1:1000 (WB) |
|  | GK600710 | Gene Tech | China | 1X (IHC) |

Figure S1


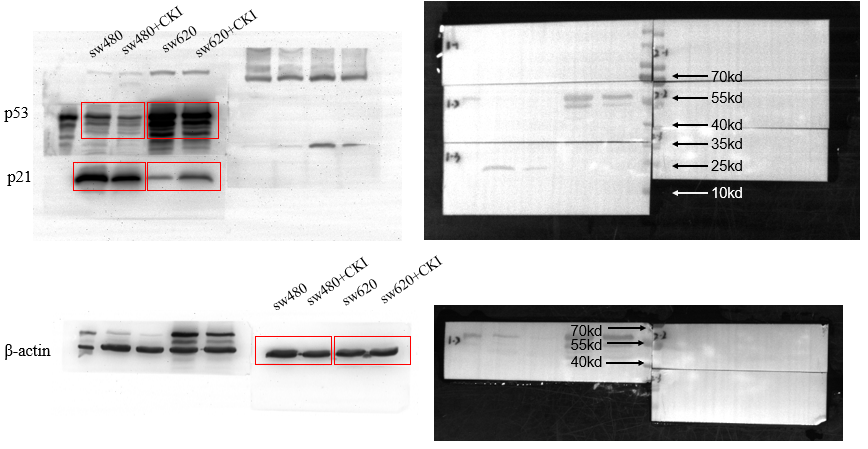


**Figure S1.** **Full length western blot of Figure 8c, 8d**

Figure S2


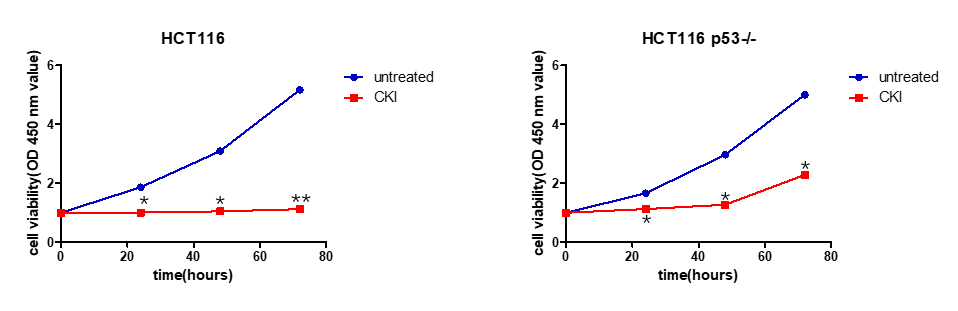


**Figure S2. Compound Kushen injection inhibits** **p53 wt and p53 null colorectal cancer cell lines proliferation *in* *vitro.*** HCT116 and HCT116 p53-/- cell viability was measured under different treatment conditions using CCK-8. Each data point represents the mean ± SD from three independent experiments. **p* < 0.05, ***p* < 0.01, compared with control.

Figure S3


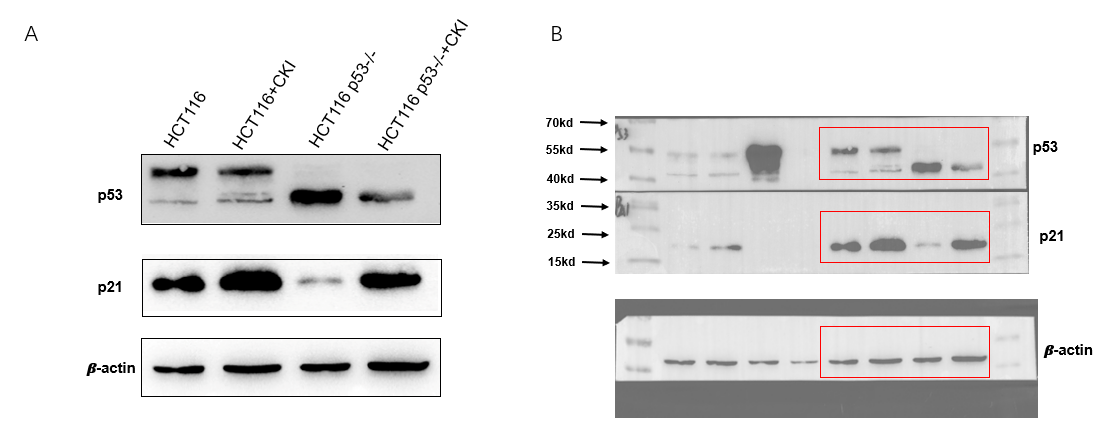


**Figure S3. Compound Kushen injection alters the expression of p21 in p53 wt and p53 null colorectal cancer cell lines.** ***A***. Western blot analysis of p53 and p21 in HCT116 and HCT116 p53-/- cells. ***B***. Full length western blot of p53 and p21 in HCT116 and HCT116 p53-/- cells.

Figure S4


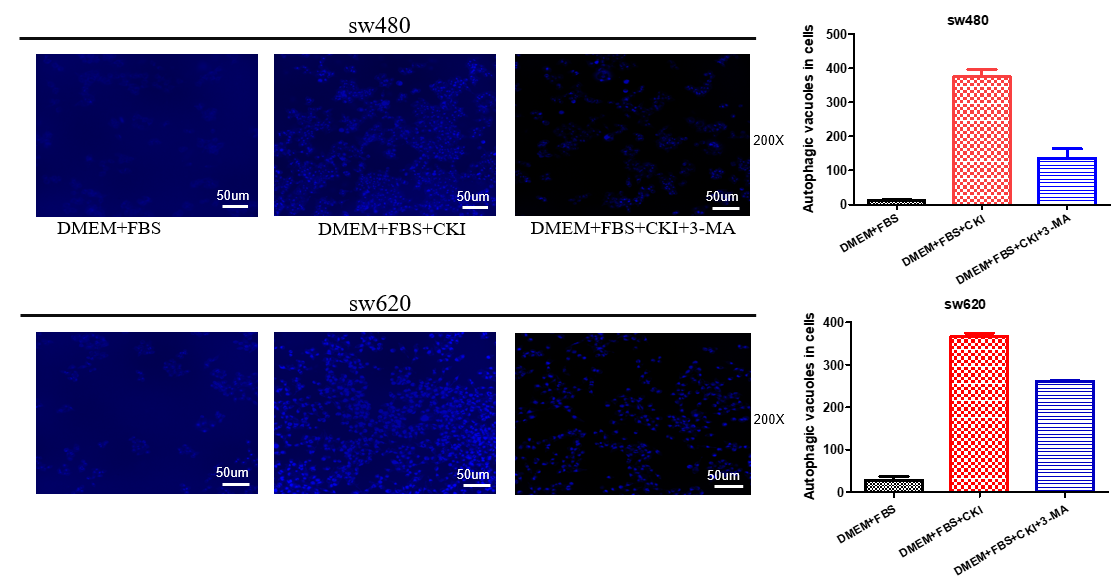


**Figure S4.** **Compound Kushen injection induces autophagy of SW480 and SW620 CRC cells and the cell death inhibited by the inhibitor 3-MA *in vitro*.** SW480 and SW620 cells were incubated in regular DMEM as a negative control. After incubated for 24h, control cells, CKI-treated cells and 3-MA-treated cells were incubated with a working solution of Autophagy Blue for 30 min at 37°C in a 5% CO_2_ incubator and then washed four times with wash buffer. Cells were imaged under a fluorescence microscope with a DAPI channel. Autophagy is indicated by the bright blue dot staining of autophagic vacuoles. Each data point represents the mean ± SD from three independent experiments.

Figure S5


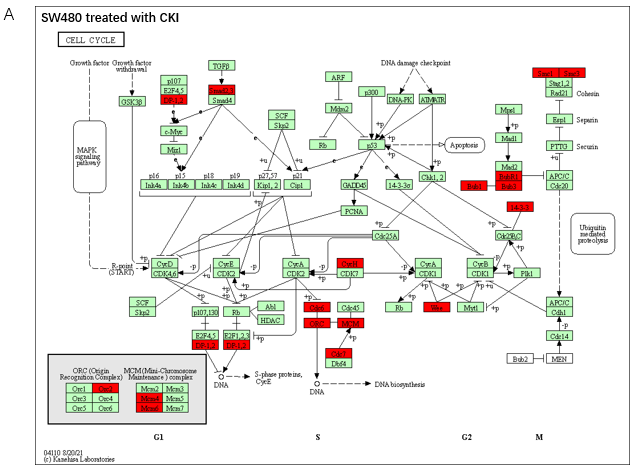


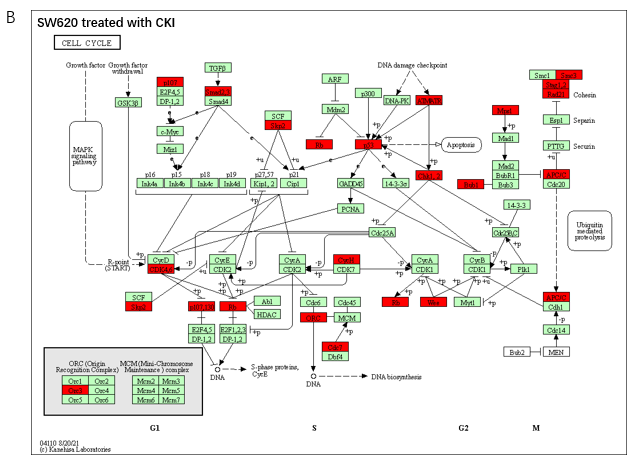


**Figure S5.** **The cell cycle pathway map showed the distribution of differentially regulated genes of RNA-Seq. *A.*** The regulation of CKI in the SW480 cells. ***B.*** The regulation of CKI in the SW620 cells. Red colors represent the targets of CKI on the cell cycle pathway.

Figure S6

A


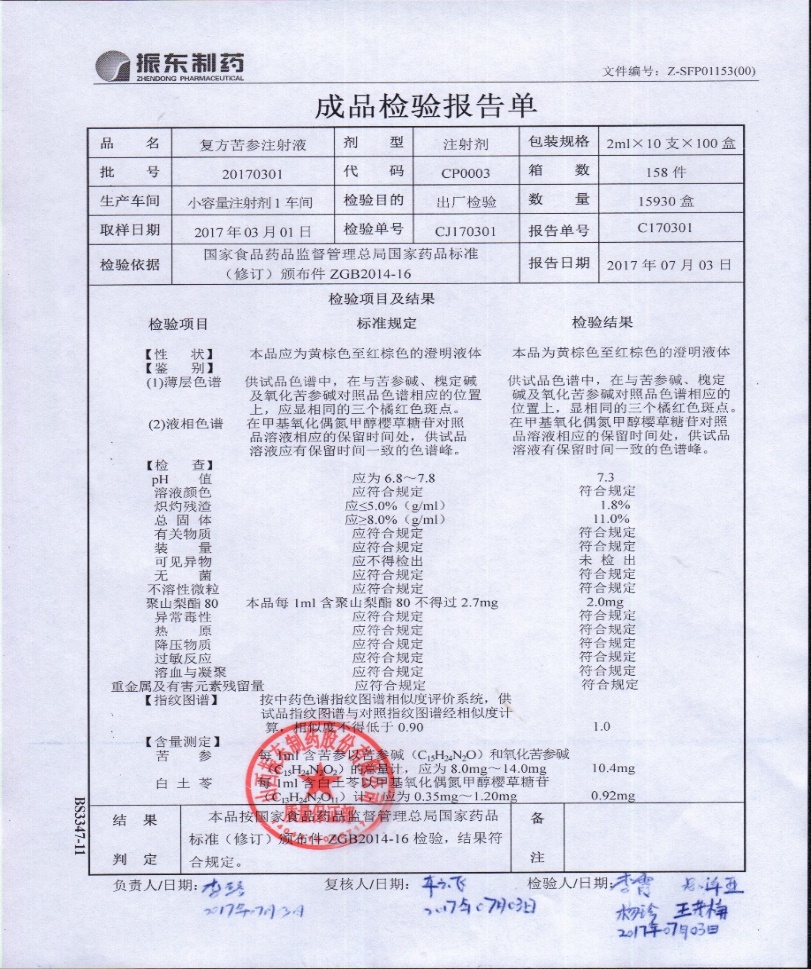


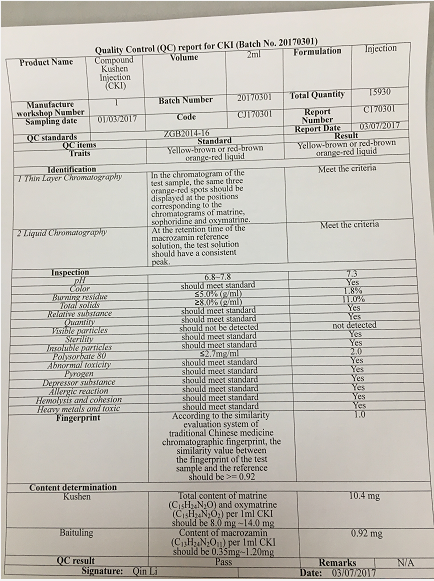


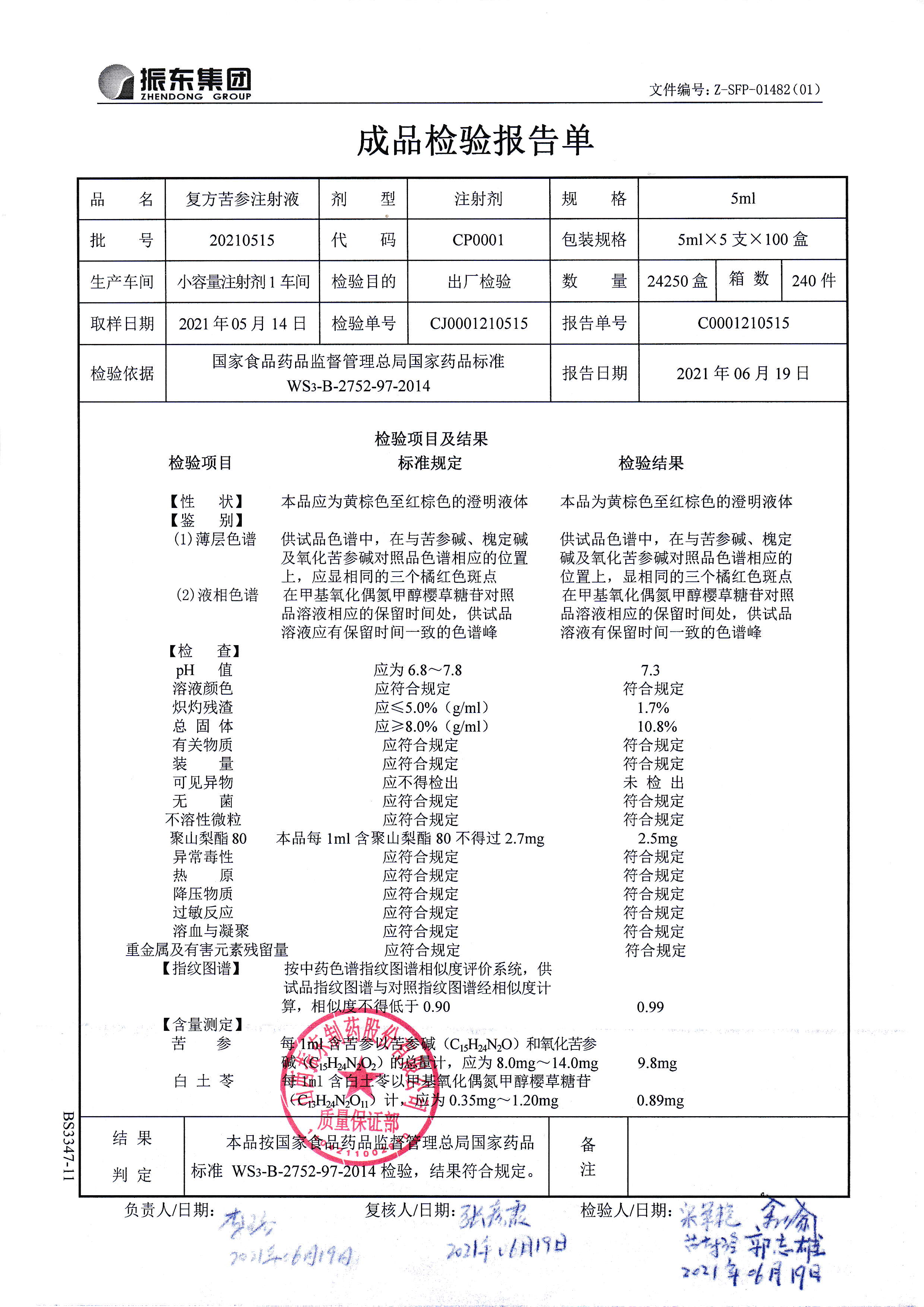


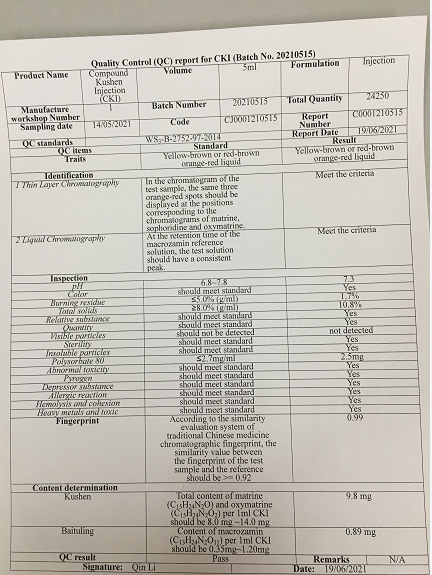


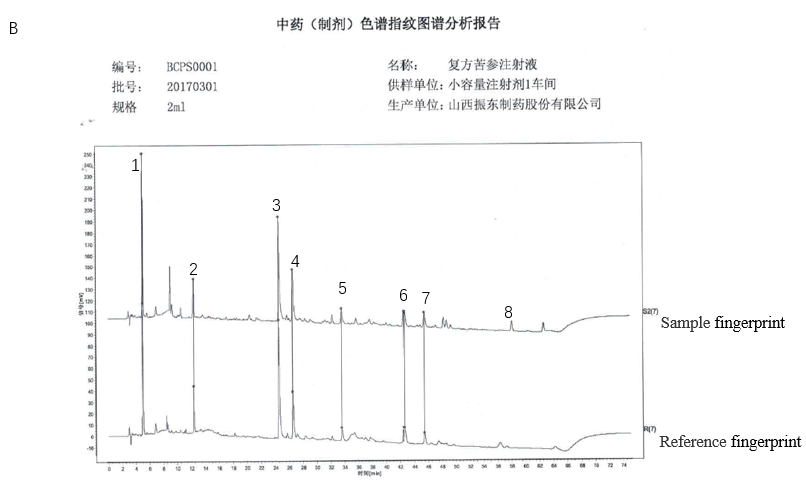


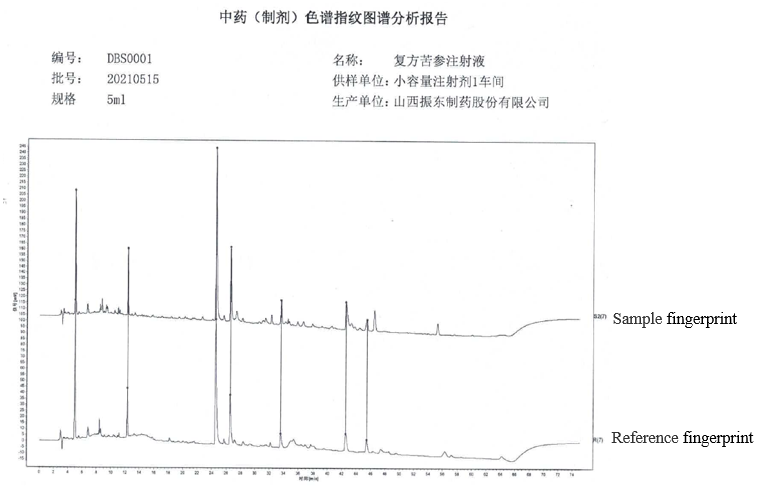


**Figure S6.** **Quality control report and fingerprint of CKI.** ***A.*** Quality control report for Batch No. 20170301 and 20210515. ***B.*** Representative fingerprint of CKI for Batch No. 20170301 and 20210515. Peak 1 is Piscidic acid, Peak 2 is Macrozamin, Peak 3 is Oxymatrine, Peak 4 is Oxysophocarpine, Peak 5 is N-methylthyramine, Peak 6 is Matrine, and Peak 7 is Sophocarping.
